# Development and psychometric validation of a novel, self-report visual processing questionnaire (ViPro-SR) for neurodivergent adults

**DOI:** 10.1007/s12144-026-09409-7

**Published:** 2026-04-28

**Authors:** Fiona Rattray, Nora Uglik-Marucha, Jacqueline Nonweiler, Dorota Ali, Michael Absoud, Francesca Happé

**Affiliations:** 1 Social, Genetic and Developmental Psychiatry Centre, King’s College London, London, UK; 2 Psychometrics and Measurement Lab, Biostatistics and Health Informatics Department, Institute of Psychiatry, Psychology and Neuroscience, King’s College London, London, UK; 3 Newcomen Neurodevelopmental Centre, Guy’s and St Thomas’s NHS Trust, London, UK

**Keywords:** Sensory sensitivity, Visual processing, Autism, ADHD, Psychometrics

## Abstract

Differences in visual sensory sensitivity/reactivity are documented in autism and ADHD. Measuring these differences could guide interventions and accommodations, improving quality of life. No current self-report questionnaires focus specifically on the range of affective and behavioural responses to visual stimuli reported in autism and ADHD. This study aimed to develop such a measure (ViPro-SR) for neurodivergent adults. Using online survey responses, psychometric properties of ViPro-SR were assessed, including factor structure, gender-related measurement invariance, internal consistency reliability and validity. An 11-item, stable 3-factor solution was derived, with factors representing: hypersensitivity to contrast, detail focus, and peripheral vision activation. Internal consistency reliability was satisfactory for the total scale (ω = 0.85) and each subscale (ω > 0.70). Evidence towards convergent validity of ViPro-SR was provided through highly significant, moderate to strong correlations with existing sensory measures. Discriminative validity was supported by significant differences in ViPro-SR total scores between autistic/ADHD groups and a not autistic/ADHD comparison group. ViPro-SR is a psychometrically sound instrument, currently recommended for subscale-level scoring, that could inform visual sensory accommodations and contribute to a research toolkit.

**Supplementary Information:**

The online version contains supplementary material available at 10.1007/s12144-026-09409-7.

## Introduction

Sensory processing refers to the ability to register and modulate sensory information and impacts how an individual interacts with their physical and social environment (Passarello et al., 2022). Although some variation in sensory processing is evident within the general population, greater differences are reported across neurodivergent conditions (NDCs). Of the different senses, visual processing, from retinal function to self-reported experiences, is of particular interest in NDCs due to myriad repercussions of altered visual sensitivity on health and cognition. However, to our knowledge, current questionnaires do not capture the range and specificity of visually driven responses and experiences, in autism and attention deficit hyperactivity disorder (ADHD).

Up to 94% of autistic individuals experience differences in perception of sensory information (Crane et al., 2009) compared to 5–14% of the general population (Ahn et al., 2004; Ben-Sasson et al., 2009). Atypical sensory responses are also more frequently reported in children (Ghanizadeh, 2011), adolescents (Fabio et al., 2024), and adults with a formal attention deficit hyperactivity disorder (ADHD) diagnosis (Bijlenga et al., 2017; Jurek et al., 2025) and with subthreshold traits (Panagiotidi et al., 2018). Differences occur across sensory systems but may be more pronounced in specific modalities (Bang & Igelstrom, 2023). Sensory profiles are typically stable in ADHD (Ben-Sasson et al., 2017) and autism (Crane et al., 2009). However some studies suggest sensory symptoms may increase across childhood in autism and ADHD (Chen et al., 2022; Cheung & Siu, 2009), but decrease across adulthood in autistic individuals (Leekam et al., 2007).

Similarities in patterns of sensory processing differences across autism and ADHD are found (Little et al., 2018), suggesting sensory phenotypes are transdiagnostic in nature and potentially driven by shared underlying neurobiological mechanisms (Scheerer et al., 2024). Despite this overlap, heterogeneity exists in findings within and between diagnostic groups, in part driven by factors such as age, gender and intellectual ability.

Sensory hypersensitivity seems to be linked to psychological and physical well-being in NDCs. First-person accounts and proxy reports attest to the detrimental impact of sensory sensitivities on everyday function (MacLennan et al., 2022). Positive associations between sensory hypersensitivity and anxiety (physical injury fears and specific phobia) (MacLennan et al., 2020), depression (Josyfon et al., 2023) and sleep difficulties (Lane et al., 2022) are reported, with visual sensory sensitivity suggested as a driver for insomnia in autistic adults (Hohn et al., 2019). Autistic adults further tend to sensory avoidance, potentially leading to missed community/social opportunities and increased loneliness (Bagatell et al., 2022; Syu & Lin, 2018), while atypical sensory perception is linked to physical symptoms such as chronic pain (Jones & Shivamurthy, 2022), migraine (Suzuki et al., 2021) and nausea (Robertson & Simmons, 2015). Although sensory hypersensitivity is associated with increased negative emotions, sensory seeking is also related to positive emotions such as peace and joy (MacLennan et al., 2022). Unsurprisingly, within the autistic community, sensory issues are highlighted as a priority area for research and support (Autistica, 2023).

Much like in autism, atypical sensory processing is linked to increased anxiety and reduced quality of life in ADHD (Engel-Yeger & Mevorach Shimoni, 2023). Sensory sensitivity correlates with emotional dysregulation (Bruton et al., 2023) and directly contributes to the relationship between ADHD symptoms and ‘emotional lability’ (DeSerisy et al., 2019). Amplified emotional responses to (unpleasant) sensory input may increase cognitive load (Fabio et al., 2024) and in turn lead to attention problems, distractibility, and organisational difficulties characteristic of ADHD (Engel-Yeger & Mevorach Shimoni, 2023). Indeed, treating sensory issues is noted to have a positive impact on ADHD traits (Khanahmadi et al., 2023). Hypersensitivity to pain and reduced tolerance of painful stimuli are also reported in ADHD and may be attenuated by dopamine agonists/stimulant medication (Treister et al., 2015).

While differences are found across all sensory modalities in autism and ADHD, a specific focus on the visual domain is warranted for several reasons. Firstly, present-day technology is arguably better suited to studying the visual system than other sensory modalities (Hutmacher, 2019). Additionally, evidence suggests differences in processing along the visual pathway in both NDCs (Little, 2018; Redondo et al., 2019), from retinal transmission of light energy into neural impulses (I. O. Lee et al., 2022) to complex cortical processes such as face perception (Lynn et al., 2018). Measurable differences in higher order visual processes may therefore be evaluated against retinal and occipital function, to help determine underlying neurobiological mechanisms that are potentially relevant across sensory systems.

Indeed, differences in visual perceptual sensitivity are noted in autism and ADHD, including colour discrimination (Banaschewski et al., 2006; Zachi et al., 2017), contrast sensitivity (Dönmez et al., 2020; Little, 2018) and motion perception (Kröger et al., 2014; Robertson et al., 2014). Autistic individuals show reduced global integration of visual information (Booth & Happé, 2018) alongside a detail-focused processing style (Happe & Booth, 2008). Pupillometry studies indicate altered latency of the pupillary response in autism (de Vries et al., 2021) and reduced task-related pupil size in ADHD (Wainstein et al., 2017), suggesting atypical physiological reactivity to visual stimuli in both NDCs, although the evidence is mixed (Bellato et al., 2020). Shared, neurobiological mechanisms such as an excitation-inhibition imbalance in neural signalling, may underlie these varied visual processing differences, differentially affecting foveal and peripheral vision (Takarae & Sweeney, 2017). Together these findings indicate that atypical processing of visual stimuli occurs across all levels of the visual pathway in both NDCs.

To trace a pathway from visual sensory experience to underlying neurobiological mechanisms, quantitative measures are needed that capture the range and extent of atypical emotional and behavioural reactivity to visual stimuli. Such measures could also form part of potential screening toolkits for NDCs in clinical contexts, and guide adaptations for individuals with sensory challenges.

Current sensory questionnaires gather information on sensory reactivity across multiple sensory modalities, but to the best of our knowledge, do not focus on one single modality in depth. By nature, this precludes capturing the breadth of atypical affective/behavioural reactivity to visual stimuli that is evident in the literature. Further, most instruments have not been developed to measure sensory patterns unique to autism or ADHD and have been standardised in neurotypical children (Ausderau et al., 2014); indeed a recent systematic review found significant limitations across sensory questionnaires that exist for use in autism (Gunderson et al., 2023).

Thus our aim was to develop two versions of a visual processing questionnaire that measures the range of affective and behavioural responses to everyday visual stimuli in NDCs - a parent/carer report version for children and young people (ViPro) and a self-report version for adults (ViPro-SR).

More specifically, we aimed to:

- develop a self-report questionnaire that successfully identifies and measures visual sensory experiences in autistic/ADHD adults and young people.
- examine the psychometric properties of the newly developed questionnaire, including its factor structure, measurement invariance due to gender, reliability, and associations with previously validated measures of ADHD/autism traits and sensory processing differences.

## Methods

The Contemporary Psychometrics Checklist (ConPsy) (Vitoratou, 2023) (temporarily withdrawn for updating) was adhered to for establishing the analysis plan. The scale was developed between February 2022 and January 2024.

### Scale development

The ViPro-SR was developed in two phases. Phase 1 involved development of an item pool and phase 2 comprised psychometric refinement and validation.

## Phase 1: Item development

Item development for the ViPro-SR was based on pilot work on a parallel parent/carer-report version (ViPro) for measuring behavioural and affective responses to visual stimuli in neurodivergent children. The research team drew on their clinical and research experience of working with neurodivergent individuals and relevant journal articles to develop an initial pool of items for ViPro. A research team member posted on Twitter (renamed X) a request for autistic individuals to provide examples of visual experiences that might be special to autism, with approximately 30 responses further informing item development. Based on these responses, items were developed under the assumption that the reported visual experiences reflected independent, largely unobservable yet real causal mechanisms (Koopmans & Schiller, 2022). The items were subsequently organised into hypothesised dimensions of visual processing (see Supplementary Table 1). These dimensions were identified pragmatically through a combination of inductive and deductive reasoning (Proudfoot, 2023), informed by synthesising evidence from clinical, academic and expert by experience sources, within a critical realist framework.

During psychometric validation of ViPro, items were refined in consultation with nine parents of neurodivergent and/or neurotypical children and young people, in line with FDA and PROMIS guidelines. Five academic and clinical experts in the field (from disciplines including neurodisability, paediatrics and occupational therapy) were also consulted to ensure content validity and usability.

Psychometric investigations of the parent/carer 18-item version suggested 14 psychometrically sound items, which clustered into three themes/aspects of visual processing – hypersensitivity to contrast, detail focus and peripheral vision activation (Lead Author, thesis), although further psychometric investigation is required. These items were used to inform development of ViPro-SR and adapted to ensure the wording was appropriate for an adult self-report questionnaire.

Two autistic adults (one with co-occurring ADHD) reviewed the items and provided feedback on their relevance and comprehensibility, paying attention to the reading level required, use of jargon, and any ambiguity (E.-H. Lee et al., 2022). For some items, two differently worded versions were given, and the autistic reviewers were asked to select their preferred version. Taking feedback into consideration, item versions were selected and 12 were modified. Both adults also piloted a semi-structed interview about autistic visual experiences, and their responses, along with further review of the literature (e.g. MacLennan et al., 2022; Parmar et al., 2021; Rattray et al., 2025) and reflection on excluded ViPro items, lead to inclusion of four additional items, resulting in an 18-item scale (see Supplementary Table 2 for the 18-item version). Wording and applicability of items was reviewed by the study team, which included researchers and health practitioners experienced in working with neurodivergent individuals. The response scale for all items was a six-point ordinal scale from ‘Never’ (= 0) to ‘Always’ (= 5) (see Supplementary Tables 2 and 3 for all response options).

## Phase 2: Psychometric refinement and validation

### Sample

Participants were recruited between June 2023 and October 2023 for an online survey study exploring workplace burnout in neurodivergent and neurotypical adults. Inclusion criteria comprised being: 18 years old or older; UK resident; and with current or previous experience of having a job. Participants who endorsed having a neurodivergent condition were asked to indicate in the survey whether this was diagnosed or self-identified. We included self-identifying individuals to maximize opportunities to gather the perspectives of historically underdiagnosed groups such as women and older age adults (e.g. O’Nions et al., 2023). As participants were anonymous, there was no formal check of diagnostic status.

Recruitment took place via networks supporting neurodivergent communities including the Autistica Discovery Network and Abilitynet. Social media (e.g. Twitter/X), King’s College London circulars and the Autism Research Centre, University of Cambridge, were also engaged for recruitment of both neurodivergent and neurotypical participants.

Participants were required to read a participant information sheet about the study and to complete a consent form before starting the online survey, hosted on Qualtrics. Ethical approval was granted by King’s College London (HR/DP-22/23–34997) and the study was performed in accordance with the Declaration of Helsinki.

Of a total of 702 people who engaged with the survey, 260 were removed for reasons including not giving full consent, not submitting the full survey or not attempting any of the ViPro-SR questions. Qualtrics bot detection software was used to identify and eliminate any automated responses. After data cleaning, the final sample size comprised 442 participants. From this sample, the overall missing value for ViPro-SR was 0.06% and the pattern of missing values appeared random. Person-mean imputation was conducted for missing values prior to testing convergent and discriminative validity of the ViPro-SR 11-item final version of the measure.

Most participants were cis gender women (67% of total sample), aged 35–54 years (48%), white (88%) and held a bachelor’s degree or higher (80%). There were significant differences between groups for: gender χ^2^ (6, *N* = 442), 21.82, *p* < 0.001; age, χ^2^ (6, *N* = 442), 26.85, *p* < 0.001; ethnicity, χ^2^ (3, *N* = 437), 17.15, *p* < 0.001 and level of education χ^2^ (3, *N* = 441), 8.09, *p* < 0.044 (see Supplementary Table 4 for sample demographic information). The comparison group comprised participants who were not autistic and did not have ADHD but could have an alternative neurodivergent diagnosis such as dyslexia (see Supplementary Table 5 for reported neurodivergent and mental health conditions within each group).

Significant differences in the expected direction were found using one-way ANOVA with Welch’s *F* correction between diagnostic groups on their corresponding trait measures (*F*(3, 136.11) = 131.04, *p* < 0.001 for RAADS-14 and *F*(3, 151.10) = 97.89, *p* < 0.001 for ASRS-5) and revealed uncorrected large effect size (η^2^ = 0.52 and ⍵^2^ = 0.52 for RAADS-14; η^2^ = 0.39 and ⍵^2^ = 0.38 for ASRS-5). Both clinically diagnosed and self-identified autistic/ADHD groups obtained mean scores on the RAADS-14/ASRS 5 that were above the recommended clinical cut-off(s) of 14, suggesting a positive screen for autism/ADHD in both formally diagnosed and self-identified groups.

### Measures

In addition to the ViPro-SR, two sensory measures were administered to evaluate convergent validity. These were the visual component from the Adolescent/Adult Sensory Profile (AASP) (Brown, 2002) and the Sensory Perception Quotient (SPQ) (Tavassoli et al., 2014). We anticipated moderate, significant correlations between the ViPro-SR and the SPQ, and between the ViPro-SR and AASP, based on their associations with other sensory measures (Skocic et al., 2024; Tavassoli et al., 2014). Although the visual component from the AASP does not form a standalone scale, this was selected for convergent validity assessment due to the absence, to the best of our knowledge, of other sensory sensitivity questionnaires dedicated to vision alone.

The visual component from the AASP contains 10 items with a 5-point ordinal scale ranging from 1 (almost never) to 5 (almost always). In our sample, the internal consistency of the AASP visual component was low (⍺ = 0.57). However, it is noteworthy that reliability of AASP items has been tested within quadrants (low registration, sensory seeking, sensory sensitivity and sensation avoiding) and not within sensory domains (e.g. auditory or visual) (Pearson, 2019). While internal consistency was acceptable for the six visual items that relate to sensory sensitivity and sensation avoiding (⍵ = 0.76), it was low for the four items associated with low registration and sensory seeking (⍺ = 0.29) (NB: it was not possible to calculate omega for the 10-item AASP visual component or for the 4-items corresponding to low registration and sensory seeking due to negative or zero item covariances, thus alpha was reported). We viewed the AASP sensory sensitivity/sensation avoiding items as having a closer theoretical relationship with ViPro-SR than the low registration/sensory seeking items. The 35-item SPQ is rated on a 4-point ordinal scale with responses ranging from 0 (strongly agree) to 3 (strongly disagree) (thus a lower score indicates higher sensory sensitivity) and had excellent internal consistency in our sample (⍵ = 0.94).

To provide evidence on the nomological network of ViPro-SR, autism and ADHD trait measures were administered, in line with evidence that visual processing differences are more common in autism e.g. (Perna et al., 2023; Simmons et al., 2009) and ADHD (Bellato et al., 2023). These measures included the Ritvo Autism and Asperger Diagnostic Scale - abridged version (RAADS-14), a 14-item self-report measure using a 4-point ordinal scale, which showed high internal consistency in our mixed neurodivergent and neurotypical sample (⍵ = 0.90) (Eriksson et al., 2013), and the Adult ADHD Self-Report Screening Scale for DSM-5 (ASRS-5), a 6-item, 5-point ordinal scale (⍵ = 0.74) (Ustun et al., 2017). We expected weak to moderate significant correlations between the ViPro-SR and the RAADS-14 and between the ViPro-SR and ASRS-5 based on previous studies that have reported significant associations between sensory measures and autistic or ADHD traits (Bang & Igelstrom, 2023; Panagiotidi et al., 2018; Taylor et al., 2020).

### Dimensionality

Exploratory factor analysis (EFA) was used to examine the underlying latent structure of ViPro-SR, followed by confirmatory factor analysis (CFA) to test the solution derived from EFA. Our sample was randomly split into two halves, with one half used for EFA and the other for CFA. To assess the suitability of our data for dimensionality investigations, we used Kaiser-Meyer-Olkin (KMO) test for sampling adequacy (Kaiser, 1970; Kaiser, 1974) and Bartlett’s test of sphericity (Bartlett, 1951). We considered a KMO value above 0.6 (Tabachnick et al., 2013), and a significant result from Bartlett’s test, as indicators of data suitability for factoring. Mardia’s (1970) multivariate skewness and kurtosis coefficients revealed a deviation from multivariate normality (*p* < 0.001) (Mardia, 1970). Consequently, we opted for maximum likelihood estimation with robust standard errors (MLR) (Asparouhov & Muthen, 2005) as the estimation method for both EFA and CFA. To determine the number of factors to retain, we employed three criteria: Kaiser Guttman criterion (Guttman, 1954; Kaiser, 1960), parallel analysis criterion (Horn, 1965), and exploratory graph analysis (EGA) (Golino & Epskamp, 2017). The results of the first two criteria were visualized using Cattell’s scree plot (Cattell, 1966). For Kaiser-Guttman criterion, the number of factors to retain corresponded to the number of eigenvalues greater than 1, whereas parallel analysis involved comparing the number of the sample eigenvalues that are larger than the mean of eigenvalues generated from 1,000 simulated random samples of the same size and number of variables. In exploratory graph analysis, a network structure was derived from the Gaussian Graphical Model (GGM) (Lauritzen, 1996) using the graphical least absolute shrinkage and selection operator on extended BIC criterium (EBICglasso) to estimate the partial correlation network. The walktrap algorithm (Pons & Latapy, 2006) was then applied to the estimated network to identify the number of communities, corresponding to the underlying factors in the data. The number of communities indicated the number of factors to retain. The solution suggested by these criteria was compared to the solutions with one more and one less factor to determine the most interpretable solution (Lim & Jahng, 2019).

In EFA, we used oblimin (oblique) rotation as we expected the factors to correlate. We considered removal of items with low primary loadings (λ$\:\le\:$0.5) and strong cross loadings (λ ≥ 0.3). For the latter, the minimum difference that we allowed between the primary factor loading and alternative factor loadings was 0.2. We also considered the solution in terms of parsimony and interpretability, ensuring a minimum of 3 items per factor and a balanced distribution of items across factors. This approach prevented the questionnaire disproportionately representing any single aspect of the construct. Additionally, we assessed how well each item’s relationship with its respective factor aligned with our theoretical expectations. Several goodness of fit indices were employed to evaluate the absolute and relative fit of the model, namely the root mean squared error of approximation (RMSEA < 0.08 indicates adequate fit and < 0.05 suggests close fit (Browne & Cudeck, 1992; Steiger, 1990); with the corresponding 90% confidence intervals, the relative chi-square (rel $\:{\chi\:}^{2}$ values < 2 and 3 indicate close and adequate fit, respectively (Hoelter, 1983; Hu & Bentler, 1999); the Tucker-Lewis Index (TLI > 0.9 indicates acceptable fit and > 0.95 close fit (Hu & Bentler, 1999; Tucker & Lewis, 1973); the comparative fit index (CFI > 0.9 and 0.95 suggest adequate fit and close fit, respectively (Bentler, 1990; Hu & Bentler, 1999); and the standardised root mean squared residual (SRMR – values below 0.05 suggest close fit and adequate fit is indicated by values below 0.08 (Hu & Bentler, 1999; Kline, 2016).

Once a suitable EFA solution was found, we tested this model using CFA in the other random half of the sample. We considered primary loadings above 0.5 as satisfactory and fit of the model to the data was evaluated based on the aforementioned criteria. After establishing the final factor structure, a bifactor model was additionally fitted, in which all items loaded on a general latent factor, while also loading on specific factors that were uncorrelated with the general factor (Holzinger & Swineford, 1937). This approach allowed us to examine whether the ViPro-SR items reflect both a general factor of visual processing and more specific subdomains, thereby assessing its potential to be used as both a multidimensional and unidimensional measure. This would provide support for using not only subscale scores but also a total score representing the broader construct of visual processing.

Dimensionality investigations were complemented by non-parametric bootstrap exploratory graph analysis (bootEGA) (Christensen & Golino, 2021) on the final set of items to investigate the stability and robustness of factors and item allocation identified from exploratory graph analysis across different subsamples. The non-parametric bootstrap exploratory graph analysis involved resampling to generate 1,000 random subsamples from the original dataset. For each of these 1,000 bootstrap samples, regularized partial correlations were estimated and their median across the samples computed to produce a median network structure. The walktrap algorithm (Pons & Latapy, 2006) was then applied to the median network to determine the number of dimensions. Structural consistency and item stability measures were then computed, with structural consistency indicating how frequently each dimension was exactly recovered (that is, having the same item composition) from the bootstrap samples, and item stability indicating how often each item was allocated in each factor.

Following literature recommendations for sample size in latent variable modelling, we aimed for a subjects-to-variables (STV) ratio of 10:1 for both EFA and CFA (Kyriazos, 2018). Since the preliminary version of ViPro-SR contained 18 items, we estimated that a sample size of at least 360 participants would be ideal. Therefore, our study’s sample size of 442 was sufficient for both EFA and CFA.

### Measurement invariance

Measurement invariance assesses whether a latent construct of a tool is measured equivalently across different groups. A measurement invariant tool ensures that observed group differences reflect genuine differences in the construct, rather than methodological bias of a measure. We evaluated the measurement equivalence of ViPro-SR due to gender using multiple group CFA (Jöreskog, 1971). We fitted and compared a series of four hierarchical invariance models (Widaman & Reise, 1997): configural, metric (weak), scalar (strong) and residual (strict), each testing for specific parameters (factor loadings, intercepts and residual variances) and progressively imposing stricter criteria than the previous one. Each level of measurement invariance was supported if changes in the fit between two nested models did not exceed recommended thresholds, with a decrease in CFI and an increase in RMSEA of 0.01 and 0.015 or greater, respectively, indicating a lack of support for measurement invariance (Chen, 2007).

Due to sample size requirements for multiple group CFA, which recommend a minimum of 100 participants per group (Kline, 2016), the analysis was limited to women and men, excluding gender-diverse participants. For the same reason, measurement invariance with respect to diagnostic group could not be evaluated, as the ADHD and control groups did not meet the required sample size thresholds.

### Reliability analysis

We evaluated internal consistency of the ViPro-SR within each factor using McDonald’s omega (⍵) (McDonald, 1999). While Cronbach’s alpha remains the most widely used index of reliability, from a factor-analytic perspective it corresponds to a restrictive model that assumes unidimensionality, equal factor loadings, and uncorrelated errors, with these assumptions seldom met in practice. McDonald’s omega does not impose these constraints and is therefore recommended as a more robust measure of internal consistency (Hayes & Coutts, 2020). For this reason, omega was employed in the reliability analysis of the present study. A value of $\:\ge\:$0.70 for ⍵ was considered satisfactory (Hair, 2019). We also examined additional indices of internal consistency for each item, including item-total correlations (ITC) and the omega if item deleted (OID). Items with ITC values in the range of 0.3 to 0.8 were considered satisfactory, indicating that they were related to the construct without being redundant. Items with OID values higher than the total subscale’s omega were identified as problematic, as they decreased the scale’s reliability.

### Validity

The concurrent convergent validity of the ViPro-SR was examined through correlations of the new measure first with total AASP scores, then with sensory sensitivity and avoidance visual items (6) and subsequently with items relating to low registration and sensory seeking (4) (Brown, 2002). The ViPro-SR was also correlated with scores on the SPQ (Tavassoli et al., 2014). For scales with less than satisfactory internal consistency (i.e., the AASP total score and the AASP visual hyposensitivity score), disattenuated correlations were additionally computed using ⍺, allowing us to examine the correlations as if the measures were perfectly reliable.

Discriminative validity was explored by determining whether scores on the ViPro-SR were significantly different across groups (i.e. between neurodivergent and comparison group participants or between autistic participants and participants with ADHD), using parametric (t-test and ANOVA) and non-parametric (Mann-Whitney and Kruskal-Wallis) test methods subject to data normality. Receiver operating characteristic (ROC) analyses were used to determine the sensitivity and specificity of ViPro-SR to discriminate between diagnostic groups (autistic versus comparison, ADHD versus comparison, autistic versus ADHD). The optimal cut-point for classifying the most individuals correctly was considered the point where the difference between sensitivity and specificity values was minimum and the sensitivity and specificity were equally high (Unal, 2017).

To provide evidence on the nomological network of the measure, we evaluated the associations of ViPro-SR with scores on measures of NDC traits: The RAADS-14, and ASRS-5.

The analyses were conducted in IBM SPSS Statistics (Version 29), R version 4.3.1 (R Core Team, 2023), and M*plus* 8 (Muthén & Muthén, 1998-2017). EFA, CFA and multigroup CFA were run in M*plus* 8. EGA and bootEGA were conducted using the R package *EGAnet* (Golino, 2024). Descriptive statistics, reliability, and validity assessments were run in IBM SPSS Statistics (Version 29).

## Results

### Dimensionality

**Fig. 1:**
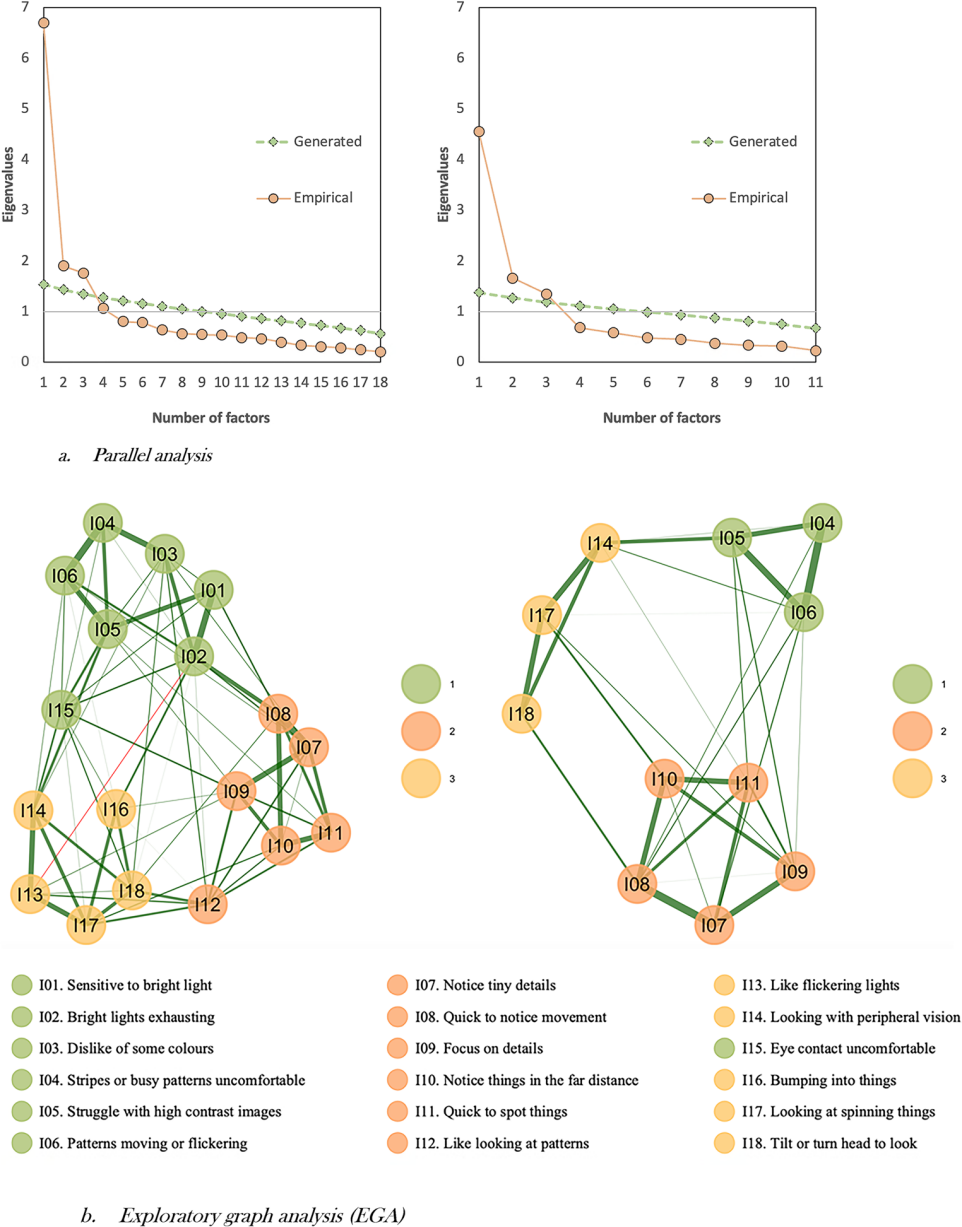
Parallel analysis (**A**) and EGA (**B**) visualisations for the 18-item (left) and 11-item (right) measure Note: In EGA, circles (nodes) represent items, and lines (edges) represent partial correlations, with edge thickness indicating the magnitude of these correlations. Green edges denote positive correlations, red edges denote negative correlations, and node colours indicate factors: green nodes represent Factor 1 (hypersensitivity to contrast), orange nodes represent Factor 2 (detail focus), and yellow nodes represent Factor 3 (peripheral vision activation)

The data was found to be suitable for factor analysis (KMO = 0.980, Bartlett’s Test of Sphericity $\:{\chi\:}^{2}$=69772.861, df=153, *p* < 0.001). EFA was conducted on one randomly split half of the data, including all 18 items (*N* = 220). Both parallel analysis and EGA (see Fig. 1) suggested retaining up to three factors, whereas the Kaiser-Guttman criterion (four eigenvalues above 1: 6.7, 1.9, 1.8, and 1.06) indicated up to four factors. However, the fourth eigenvalue (1.06) was considerably lower than the third (1.8). Examining the four-factor solution revealed that one factor had fewer than the minimum required number of items with meaningful loadings, leading to the investigation of a three-factor solution.

The three-factor solution showed an acceptable fit (rel $\:{\chi\:}^{2}$ =2.04; RMSEA = 0.069 [90% CI: 0.055–0.082]; TLI = 0.897; CFI = 0.931; SRMR = 0.038). However, some items either had substantial cross-loadings (λ ≥ 0.3) on other factors (I01, I02, I12) or did not load strongly (λ ≤ 0.5) on any factor (I16, I15, I03), and were sequentially removed from the model. The resulting 12-item, three-factor solution showed a close fit to the data (rel $\:{\chi\:}^{2}$ = 1.56; RMSEA = 0.050 [90% CI: 0.020–0.076]; TLI = 0.961; CFI = 0.981; SRMR = 0.024).

The EFA-derived solution was tested on another random split half of the data using CFA (*N* = 222). Although it showed an acceptable fit (rel $\:{\chi\:}^{2}$= 2.37; RMSEA = 0.078 [90% CI: 0.060–0.097]; TLI = 0.906; CFI = 0.927; SRMR = 0.053), item I13 had a low loading on its main factor (λ = 0.36). EFA was repeated excluding I13, with all items presenting satisfactory loadings (λ ≥ 0.50; please see Table 1) and the model having close fit to the data (rel $\:{\chi\:}^{2}$=1.77; RMSEA = 0.059 [90% CI: 0.028–0.087]; TLI = 0.953; CFI = 0.979; SRMR = 0.022). Parallel analysis, EGA and the Kaiser-Guttman criterion (three eigenvalues above 1: 4.5, 1.7, 1.3) were re-computed on this reduced set of items, still indicating that up to three factors should be retained. The final 11-item, three-factor solution was tested using CFA and demonstrated an acceptable fit (rel $\:{\chi\:}^{2}$=2.25; RMSEA = 0.075 [90% CI: 0.055–0.096]; TLI = 0.924; CFI = 0.943; SRMR = 0.049). The three factors were deemed to represent: hypersensitivity to contrast (e.g. looking at stripes or busy patterns is uncomfortable for me), detail focus (e.g. I tend to focus on details or parts of objects/scenes rather than the whole thing) and peripheral vision activation (e.g. I tilt or turn my head sideways to look at things) (see Supplementary Table 6 for items included in the final CFA solution). The author team concluded that the 11-item 3-factor solution fitted well with both parsimony and interpretability recommendations (Vitoratou, 2023).

**Table 1:** Descriptive indices and factor loadings of the final 11-item ViPro-SR measure

Item	ViPro statements (abbreviated)	Mean (SD)	Median (Q1-Q3)	Mode (min-max)	Average diagnostic group differences, (Cohen’s d [95% CI])		EFA λ (se)	CFA λ (se)
					Autism vs. ADHD^‡^	ND vs. Comp^‡^		
*Factor 1*,* hypersensitivity to contrast*,* ω [bootstrap 95% CI] = 0.854 [0.826*,* 0.878]*								
I04	Stripes or busy patterns uncomfortable	2.05 (1.51)	2 (1–3)	2 (0–5)	0.51* (0.35 [0.02, 0.67])	1.26*** (0.88 [0.62,1.14])	0.832 (0.05)	0.79 (0.04)
I05	Struggle with high contrast images	1.93 (1.55)	2 (1–3)	2 (0–5)	0.37 (0.25 [−0.07, 0.57])	1.46*** (1.00 [0.74, 1.27])	0.679 (0.07)	0.864 (0.03)
I06	Patterns moving or flickering	2.27 (1.48)	2 (1–3)	2 (0–5)	0.39 (0.27 [−0.05, 0.59])	1.15*** (0.82 [0.56, 1.08])	0.788 (0.05)	0.853 (0.03)
*Factor 2*,* detail focus*,* ω [bootstrap 95% CI] = 0.867 [0.843*,* 0.888]*								
I07	Notice tiny details	3.26 (1.33)	3 (2–4)	3 (0–5)	0.57** (0.48 [0.16, 0.81])	1.30*** (1.05 [0.78, 1.31])	0.831 (0.05)	0.82 (0.04)
I08	Quick to notice movement	3.39 (1.25)	4 (3–4)	4 (0–5)	0.30 (0.25 [−0.07, 0.57])	1.18*** (1.01 [0.74, 1.27])	0.797 (0.04)	0.859 (0.03)
I09	Focus on details	2.99 (1.25)	3 (2–4)	3 (0–5)	0.48* (0.42 [0.10, 0.74])	1.52*** (1.34 [1.07, 1.61])	0.664 (0.06)	0.715 (0.04)
I10	Notice things in the far distance	2.45 (1.35)	2 (1.75–3.75)	2 (0–5)	0.13 (0.10 [−0.22, 0.42])	1.06*** (0.82 [0.56, 1.08])	0.757 (0.05)	0.641 (0.05)
I11	Quick to spot things	2.83 (1.30)	3 (2–4)	2 (0–5)	0.53* (0.42 [0.10, 0.75])	0.98*** (0.78 [0.52, 1.04])	0.78 (0.05)	0.627 (0.05)
*Factor 3*,* peripheral vision activation*,* ω [bootstrap 95% CI] = 0.702 [0.645*,* 0.755]*								
I14	Looking with peripheral vision	0.54 (0.87)	0 (0–1)	0 (0–5)	0.16^a^ (−0.08^b^)	0.40^a^*** (0.17)	0.518 (0.1)	0.535 (0.07)
I17	Looking at spinning things	1.60 (1.36)	1 (0–2)	0 (0–5)	−0.12 (−0.09 [−0.41, 0.23])	0.91*** (0.69 [0.43, 0.95])	0.733 (0.1)	0.581 (0.06)
I18	Tilt or turn head to look	1.76 (1.35)	2 (1–2)	2 (0–5)	−0.03 (−0.02 [−0.34, 0.30])	0.79*** (0.60 [0.34, 0.86])	0.583 (0.1)	0.881 (0.05)

ω = McDonald’s omega; 95% CI: 95% confidence intervals; EFA λ (se): exploratory factor analysis loadings (standard error); CFA λ (se): confirmatory factor analysis standardised loadings (standard error). ^***‡***^ comparison via independent t tests and

ND (autistic and/or ADHD), Comp (Comparison - not autistic and not ADHD but may have a different neurodivergent condition e.g. dyslexia, or be neurotypical)

* *p* < 0.05 **p < 0.01. *** *p* < 0.001. All loadings are statistically significant, p < 0.05

^a^Mann-Whitney test; ^b^ Pearson’s r effect size

The bifactor model, which included three specific factors (‘hypersensitivity to contract,’ ‘detail focus,’ and ‘peripheral vision activation’) alongside a general ‘visual processing’ factor comprising all items, was examined but resulted in a Heywood case, with a large negative variance estimate for item I10. Because the negative variance was substantial, constraining it to zero was not advisable, and the model was deemed inadmissible, suggesting limited support for a general factor in our data. Item I10 was retained, however, as it did not show issues in either EFA or CFA. These findings support the multidimensionality of the measure, suggesting that it is most appropriate to compute subscale scores for the three specific factors rather than a general total score. If a total score is used, it should be treated only as a composite index, rather than as a reflection of a single underlying construct.

**Fig. 2:**
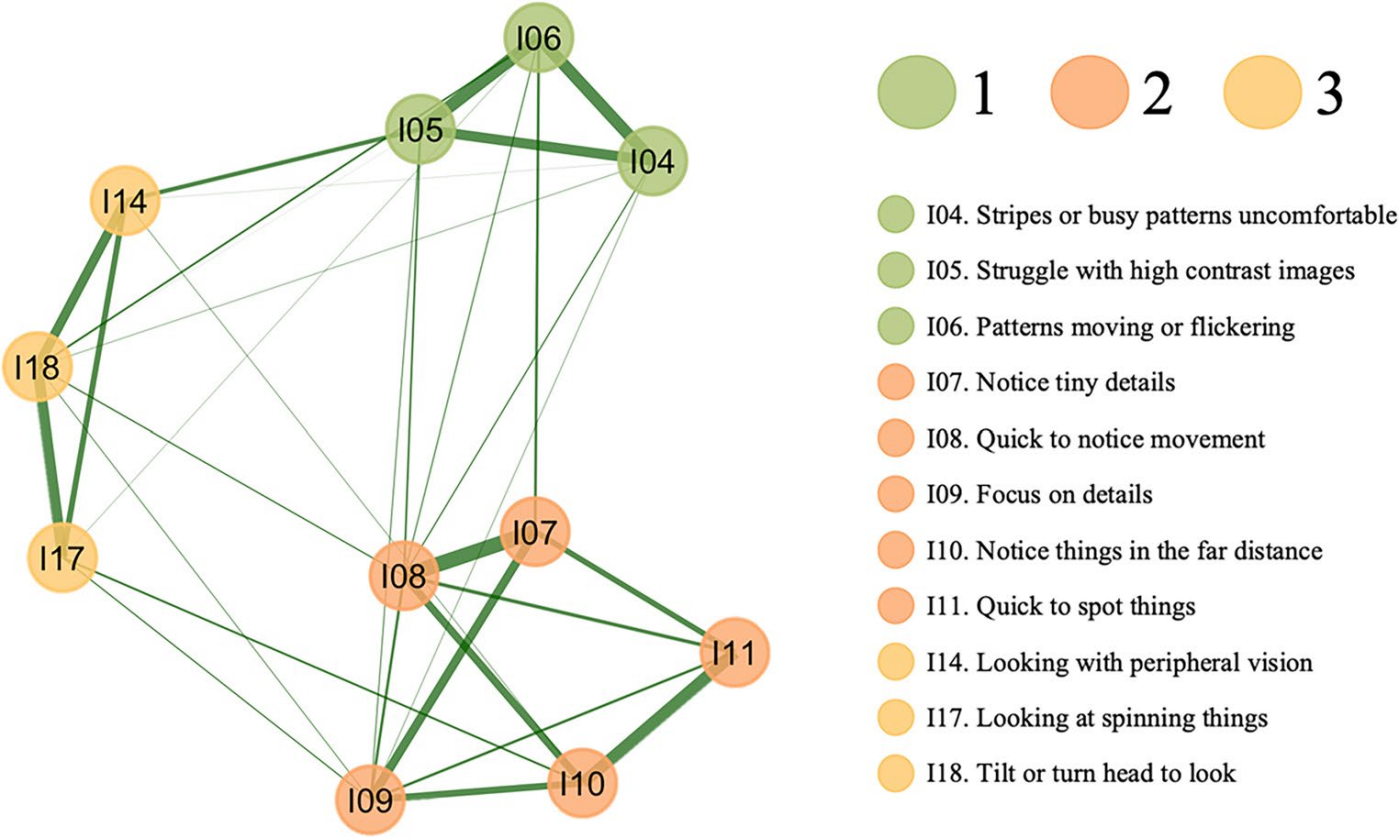
Median network structure of 11-item ViPro estimated using non-parametric bootstrap EGA with 1,000 iterations. Note: Green nodes represent Factor 1 (hypersensitivity to contrast), orange nodes represent Factor 2 (detail focus), and yellow nodes represent Factor 3 (peripheral vision activation)

The stability of the 11-item, three-factor structure of ViPro-SR was evaluated using non-parametric bootEGA on the full sample (*N* = 442), with the median network structure shown in Fig. 2. Across 1,000 bootstrap samples, the median number of factors was estimated to be three (se = 0.032, 95% CI [2.94, 3.06]). A three-factor structure was identified in 99.9% of the samples, indicating that three-factor solution to be highly stable across samples. While a two-factor solution was also identified, it was observed only 0.1% of the time. The structural consistency of each factor, that is, identical item allocation to each factor, was very high. Specifically, Factor 1 (hypersensitivity to contrast) and Factor 2 (detail focus) were exactly replicated 100% of the time across all bootstrap samples, and Factor 3 (peripheral vision activation) was replicated 99.9% of the time. An examination of the stability of each item within each factor (see Supplementary Table 7) indicated that all items consistently replicated in their respective factors at least 99.9% of the time. Items from Factor 3 (peripheral vision activation), while being consistently identified in their theoretical factor, very infrequently replicated on other factors. Specifically, items I14, I17 and I18 were allocated to their intended factor 999 times, but also replicated on Factor 1 (hypersensitivity to contrast) once.

### Endorsement

Table 1 presents descriptive statistics for the final 11 items of ViPro-SR. The items with the highest endorsement were I08 (‘quick to notice movement’), I07 (‘notice tiny details’), and I09 (‘focus on details’), with 75.3%, 72.8%, and 65.3% of participants, respectively, rating them as 3 (‘Often’) or higher. In contrast, the least endorsed statements were I14 (‘looking with peripheral vision’) and I17 (‘looking at spinning things), which were rated as 0 (‘Never’) or 1 (‘Very rarely’) by 87.1% and 51.4% of participants, respectively. Significant group differences were found for individual ViPro-SR items (see Table 2).

**Table 2:** Mean score (max = 5) and standard deviation (sd) for individual ViPro-SR items (final 11-item solution) by diagnostic group

Item	Diagnostic group			
	Autistic	ADHD	Autistic and ADHD	Comparison (not autistic or ADHD)
I04	2.22_a, b_ (1.50)	1.71_a_ (1.39)	2.50_b_ (1.42)	0.99_c_ (1.16)
I05	2.13_a_ (1.50)	1.76_a_ (1.46)	2.37_a_ (1.58)	0.70_b_ (0.97)
I06	2.41_a, b_ (1.49)	2.02_a_ (1.14)	2.71_b_ (1.43)	1.30_c_ (1.26)
I07	3.46_a_ (1.20)	2.89_b_ (1.15)	3.71_a_ (1.24)	2.17_c_ (1.30)
I08	3.50_a_ (1.18)	3.20_a_ (1.20)	3.88_b_ (1.12)	2.40_c_ (1.11)
I09	3.19_a, b_ (1.13)	2.71_a_ (1.20)	3.50_b_ (1.11)	1.71_c_ (0.97)
I10	2.57_a_ (1.28)	2.44_a_ (1.37)	2.79_a_ (1.34)	1.56_b_ (1.22)
I11	3.09_a_ (1.26)	2.56_a, b_ (1.31)	2.96_a_ (1.26)	2.01_b_ (1.11)
I14	0.65_a_ (0.96)	0.49_a, b_ (0.87)	0.54_a_ (0.85)	0.20_b_ (0.44)
I17	1.68_a_ (1.35)	1.80_a_ (1.34)	1.83_a_ (1.42)	0.83_b_ (0.99)
I18	1.79_a_ (1.43)	1.82_a_ (1.35)	2.10_a_ (1.24)	1.10_b_ (1.05)

ANOVAs and a Kruskall-Wallis test (I14) were used for group comparisons. Means with differing subscripts within rows are significantly different at the *p* < 0.05 level based on Bonferroni and Games-Howell post-hoc comparisons and all pairwise comparisons (I14)

### Measurement invariance

Measurement invariance of the final 11-item, 3-factor model of ViPro-SR was examined with respect to gender (N_men_=96, N_women_=298) for the entire sample (*N* = 394). The configural model had acceptable fit to the data (see Table 3), allowing investigation of further invariance levels. Metric (weak), scalar (strong), and residual (strict) measurement invariance were supported as indicated by ΔRMSEA and ΔCFI values (see Table 3 for comparisons). Full measurement invariance was thus established, indicating that the ViPro-SR measures visual processing equivalently across men and women, allowing for fair and meaningful score comparisons between these groups. Mean ViPro-SR total score (11-item) was significantly higher for women (*M* = 25.46, *SE* = 0.56) than men (*M* = 22.73, *SE* = 0.94), *t*(392) = 2.44, *p* = 0.015.

**Table 3:** Goodness of fit indices for nested measurement invariance models with respect to gender (N_men_=96, N_women_=298) in the entire sample (*N* = 394)^a^

Model	χ^2^	df	RMSEA (90% CI)	CFI	Model comparison	Change in model fit		Decision
						ΔRMSEA	ΔCFI	
1. Configural invariance	94.175	82	0.075 (0.06, 0.091)	0.942	-	-	-	
2. Metric (weak) invariance	181.526	90	0.072 (0.057, 0.087)	0.942	Model 2 vs. 1	−0.0030	0.000	Supported
3. Scalar (strong) invariance	200.648	98	0.073 (0.058, 0.087)	0.935	Model 3 vs. 2	0.0010	−0.007	Supported
4. Residual (strict) invariance	221.469	109	0.072 (0.059, 0.086)	0.928	Model 4 vs. 3	−0.0010	−0.007	Supported

χ^2^: chi-square; df: degrees of freedom; RMSEA (90% CI): root mean square error of approximation (90% confidence intervals); CFI: the comparative fit index. All χ^2^ p-values were < 0.0001. Rejection of invariance was based on ΔCFI > 0.010 and ΔRMSEA > 0.015 (Chen, 2007). MLR estimator was used. ^a^ Due to sample size requirements for multiple group CFA, the analysis was limited to women and men, excluding gender-diverse participants

### Reliability

Internal consistency reliability was satisfactory for both the total scale (⍵ = 0.85) and each subscale (⍵ ≥ 0.70) (see Table 1). Corrected item-total correlations within each factor fell within the range of 0.3 to 0.8, indicating that items are related to the construct without being redundant. Omega if item deleted ranged from 0.83 to 0.85 for the entire scale.

### Validity

#### Convergent validity

ViPro-SR (11-item) total score was strongly correlated with the SPQ total *r*=−0.71 [−0.75, −0.66], *p* < 0.001 and moderately correlated with the visual component total of the AASP *r*= 0.58 [0.51, 0.64], *p* < 0.001 (disattenuated: r̂=0.82), demonstrating expected findings for convergent validity (see Supplementary Fig. 1). Correlations held within diagnostic groups (see Supplementary Table 8). ViPro-SR showed a much stronger association with the sensory sensitivity and avoidance items/subdomain from the visual component of the AASP *r*=0.70 [0.65, 0.75], *p* < 0.001, than with items relating to low registration and sensory seeking, where a weak negative correlation was observed, *r*=−0.13 [−0.22, −0.36], *p* = 0.007 (disattenuated: $\:\widehat{r}$=−0.28). The three ViPro-SR factors showed moderate to strong significant correlations with the SPQ35 and the AASP total scores (all *r*≥0.40, *p* < 0.001, disattenuated correlations with the AASP were $\:\widehat{r}$≥0.63) (see Supplementary Table 9).

A significant relationship between ViPro-SR total score and SPQ35 score total across the total sample remained when controlling for autism traits with the RAADS-14 total score, *r*=−0.60, *p* < 0.001 and for ADHD traits with the ASRS-5 total *r*=−0.66, *p* < 0.001. The relationship between ViPro-SR and AASP visual component remained significant when controlling for autism traits, *r*=0.42, *p* < 0.001 and ADHD traits, *r*=0.47, *p* < 0.001.

Regarding assessment of the nomological network of the measure, ViPro-SR showed significant, moderate, positive correlations with the RAADS-14, *r*=0.63 [0.57, 0.68], *p* < 0.001 and with the ASRS5, *r*=0.45 [0.37, 0.52], *p* < 0.001.

#### Discriminative validity

Autistic adults scored significantly higher (M = 26.71, SD = 8.90) overall on the ViPro-SR than adults in the comparison group (*M* = 14.97, *SD* = 6.87), *t(*150.87) = 11.49 *p* < 0.001, with an effect of *d* = 1.39. Adults with ADHD also scored significantly higher (M = 23.40, SD = 8.90) than the comparison group on the ViPro-SR total, *t(*76.98) = 5.40, *p* < 0.001, with an effect of *d* = 1.09. The greatest difference in total scores occurred between autistic adults with co-occurring ADHD (*M* = 28.89, *SD* = 7.58) and the comparison group, *t(*181.00) = 12.51, *p* < 0.001, with a large effect of *d* = 1.90. Significant diagnostic group differences were found for each factor (see Table 4).

**Table 4:** Means and standard deviations for ViPro total and factor scores as a function of diagnostic group with group comparisons

Diagnostic group	ViPro-SR total score (11 items)	ViPro-SR Factor 1: Hypersensitivity to contrast	ViPro-SR Factor 2: Detail focus	ViPro-SR Factor 3: Peripheral vision activation
	Mean (SD)	Mean (SD)	Mean (SD)	Mean (SD)
Autistic (*n* = 214)	26.71_a, b_ (8.90)	6.77 _a, b_ (3.96)	15.82 _a, b_ (4.81)	4.13 _a_ (2.93)
ADHD (*n* = 45)	23.40 _a_ (8.90)	5.49 _a_ (3.42)	13.80 _a_ (5.15)	4.11 _a_ (2.89)
Autistic and ADHD (*n* = 113)	28.89_b_ (7.58)	7.58 _b_ (3.70)	16.84 _b_ (4.50)	4.47 _a_ (2.73)
Comparison (*n* = 70)	14.97_c_ (6.87)	2.99 _c_ (2.90)	9.86 _c_ (4.46)	2.13 _b_ (1.86)
*F*(3, 438) F (3, 151.63) F (3, 150.64)	46.12***	34.67***	36.62***	21.06***
η^2^	0.24	0.15	0.20	0.08

Means with differing subscripts (within columns) are significantly different at *p* < 0.05 based on Bonferroni (factor 2) and Games-Howell (factors 1 and 3) comparisons

***p < 0.001

Beyond diagnostic groupings, differences between the whole sample for ViPro-SR (18-items) and age band were negligible: there was not a significant effect of age-band on ViPro-SR total score F(2,435) = 0.26, *p* = 0.775, ⍵^*2*^ = −0.003 (*h2* = 0.001). ViPro-SR total scores were higher for participants who had not obtained a university degree (Bachelor’s or higher) (*M* = 45.66, *SE* = 1.63) than for those who had (*M* = 41.26, *SE* = 0.81) *t*(435) = 2.44, *p* = 0.015, with effect size *d* = 0.29. Further, there was a small, significant effect of gender on ViPro-SR total score, F(2,435) = 6.89, *p* = 0.001, ⍵^*2*^ = 0.026 (*h2* = 0.031).

There was a significant difference in the mean ViPro-SR total score [F(3, 437) = 46.43, *p* < 0.001] between groups, whilst adjusting for age band (3 groups: 18–34 years; 35–54 years; and 55–75 + years). Group differences between ViPro-SR total score also remained significant when adjusting for gender (3 groups: cisgender woman, cisgender man and non-binary/other self-described gender) [F(3, 437) = 47.31, *p* < 0.001], education level (2 groups: undergrad and post-graduate degree) [F(3, 436) = 44.31, *p* < 0.001] and ethnicity (2 groups: white and all other ethnic groups combined) [F(3, 432) = 46.02, *p* < 0.001].

**Fig. 3:**
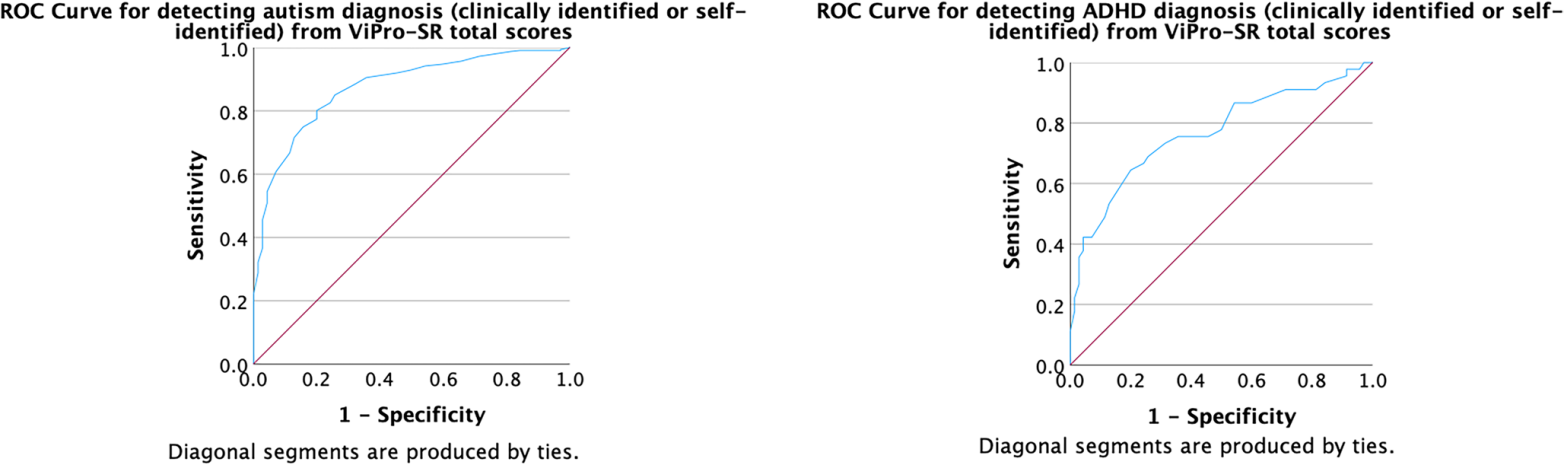
ROC curves of the sensitivity and specificity of ViPro-SR for classifying autistic participants (with or without ADHD) from comparison participants and for classifying participants with ADHD from comparison participants

Receiver operating characteristic (ROC) analysis was used to gauge the ability of ViPro-SR total score to accurately classify autistic participants (with and without ADHD) from participants in the comparison group. The area under the ROC curve (AUC) had a value of 0.87 (CI 0.83–0.92), *p* < 0.001, demonstrating good/excellent discriminatory power (Hosmer, 2013; Nahm, 2022) (see Fig. 3). At a ViPro-SR total score cut-off point of 20.5, individuals scoring at or above this threshold were 80% likely to be correctly identified as autistic. Individuals scoring below this threshold were around 80% likely to be correctly identified as not autistic.

ROC analysis found ViPro-SR to show acceptable classification of participants with ADHD (clinically diagnosed and self-identified) from comparison group participants (AUC = 0.77, CI 0.67–0.86, *p* < 0.001) (see Fig. 3). Classification of autistic participants from those with ADHD was at chance level, AUC = 0.59 (CI = 0.50–0.68, *p* = 0.056).

## Discussion

The aim of this study was to develop and evaluate a new self-report measure of visual sensory experiences (ViPro-SR) in neurodivergent adults, which can be used in English-speaking Western contexts. Co-developing the ViPro-SR items with neurodivergent adults, health practitioners, and researchers specialising in neurodiversity ensured the creation of a relevant and meaningful measure, which demonstrates sound psychometric properties. Psychometric analyses revealed an 11-item, 3-factor model consistent with a priori hypotheses, which demonstrated excellent structural consistency, indicating that the three factors are robust and likely to be reproducible across different samples from the same population. ViPro-SR demonstrated satisfactory internal consistency, strong evidence towards validity of the scale, and measurement invariance (non-bias) with respect to gender. ViPro-SR is the first validated self-report measure to capture the multidimensional aspects of visual processing characteristic of the experiences of autism/ADHD.

The three dimensions measuring aspects of visual processing pertained to hypersensitivity to contrast, detail focus, and peripheral vision activation. High stability and generalisability of the three-factor structure of ViPro-SR was found. Further, items consistently aligned with their respective factors, indicating that these dimensions are well-defined and measure distinct, yet related, aspects of visual processing. This distinctiveness ensures robustness and provides a clear, interpretable model of these visual processing dimensions. Given the strong psychometric support for the multidimensional structure of the ViPro-SR, the use of subscale-level scores is currently recommended. At present, the total score should be treated only as a composite index, a pragmatic summary measure, while further research using bifactor approaches in larger samples is recommended to provide additional support for this scoring decision.

The constructs that emerged from psychometric evaluation of ViPro-SR reflect literature concerning sensory differences in autism/ADHD. Hypersensitivity to light (photophobia) and contrasting colours are well documented in autism (Parmar et al., 2021; Robertson & Simmons, 2015) and increasingly so in ADHD (Kooij & Bijlenga, 2014) and associated with physical discomfort, including nausea and migraine (Grandgeorge & Masataka, 2016; Ludlow & Wilkins, 2009). Intriguingly, two of the three items eliminated from this factor were related to light sensitivity rather than colour contrast. While photophobia appears to be common amongst autistic individuals, it is also associated with a wide range of ocular and other health conditions (Digre & Brennan, 2012), thus may not effectively differentiate neurodivergent from neurotypical visual experiences. Conversely, although sensitivity to colour contrast is reported in the population at large, frequent discomfort from images with a large chromaticity separation is potentially more specific to neurodivergent populations. Recent studies indicate that average differences in chromaticity are a good predictor of visual discomfort from an image, and greater discomfort is associated with larger neural and haemodynamic responses in the visual cortex (Haigh et al., 2019). Discomfort is hypothesized to serve as a homeostatic mechanism to avoid stimuli that cause heightened excitation (Haigh et al., 2019). Accordingly, the threshold for discomfort may be lower in conditions that are associated with atypical cortical hyperexcitability (Bargary et al., 2015), such as migraine, epilepsy, autism and ADHD. Interestingly migraine has been reported as more common in both autism and ADHD (Lee et al., 2021; Soltan et al., 2023), and both NDCs are associated with an increased prevalence of epilepsy (Lukmanji et al., 2019; Wang et al., 2023), suggesting shared underlying mechanisms may subserve hypersensitivity to light and contrast.

Autism literature widely supports the construct of ‘detail focus’ as capturing a processing bias for featural and local information (Happe & Booth, 2008). Local visual perception may be thought of as processing restricted to smaller regions of the visual field (with little requirement for interaction between different regions) or aspects of a visual stimulus that can be processed in isolation (Van der Hallen et al., 2015). Conversely, global processing involves larger regions of the visual field and interaction between regions to form a full picture of the overall scene. A narrower functional field of view (FFoV) could account for superior local processing characteristic of autism, alongside relative difficulties with extracting global information from a scene (Song et al., 2015). Indeed, a recent study of 145 twin pairs found individuals with a clinical autism diagnosis and/or with autistic traits required more visual information for gestalt processing using the fragmented pictures test, indicative of reduced global processing (Neufeld et al., 2020). Differences in speed of processing, but not in accuracy, were identified in a meta-analysis including around 1000 autistic participants, suggesting slower and less efficient early stage of pre-attentive global processing and a later stage advantage in processing of local elements (Van der Hallen et al., 2015). A faster switch to processing details and reduced processing of gist is in line with findings that autistic people are able to integrate details globally but require more cues/time, and supports the (updated) weak central coherence (WCC) account of a local processing bias (Frith, 1989; Happe & Frith, 2006).

Peripheral vision activation, the third construct to emerge, relates to engaging and stimulating retinal regions outside the fovea and the related neural pathways. This may be achieved through side glancing or moving the head slightly to one side while keeping the eyes reasonably still. Side glancing is often alluded to in clinical and community reports. Two research studies identified increased unusual visual exploration (e.g. examining objects from odd angles or in peripheral vision) in infants subsequently diagnosed as autistic, and the authors suggested that this may be an early indicator of autism (Miller et al., 2021; Ozonoff et al., 2008). Increased lateral vision (looking out of the side of the eyes) in autism potentially reflects a conscious or subconscious attempt to facilitate processing of information in peripheral vision that requires high temporal resolution and to limit excessive information processing at the fovea (Mottron et al., 2006). Whereas central or foveal vision enables high chromatic and spatial acuity needed for everyday visual tasks such as reading (Sinha et al., 2017), peripheral vision is relied on for many other aspects of visual processing including motion discrimination (Turano & Wang, 1992), gist recognition (Larson & Loschky, 2009; Vater et al., 2022), postural control (Turano et al., 1993) and navigating around objects and people during locomotion (Peli et al., 2016); abilities found to be altered in autism and ADHD (e.g. Lim et al., 2017). Accumulating evidence suggests the balance between excitatory and inhibitory (GABA-mediated) neurotransmission is disrupted in autism (Horder et al., 2018) and ADHD (Ferranti et al., 2024; Puts et al., 2020), with potentially reduced retinal inhibition. The rod dominated peripheral retina may be uniquely affected due to its large receptive fields and subsequent reliance on inhibitory interneurons for shaping responses. Side glancing may stimulate ‘sluggish’ or altered peripheral vision by augmenting recruitment of rod dominant pathways for motion processing and by stimulating inhibitory surround mechanisms for contrast detection. Hypersensitivity to colour contrast (processed foveally) and difficulties with aspects of vision reliant on peripheral processing (e.g., perceiving the gist of a scene at a glance, or walking along a busy pavement) would therefore render increased use of lateral vision a highly adaptive behaviour when considering a need to sometimes dampen foveal input and enhance peripheral stimulation.

The amplified lateral glancing reported in ADHD may relate to increased shifts in the field of view (FoV) away from targets (Mangalmurti et al., 2020). Eye-tracking studies have found that children with ADHD struggle to maintain visual focus on a target and make excessive off-target eye movements or saccades (Mangalmurti et al., 2020). Pervasive under-arousal or dysregulated arousal in ADHD is proposed to lead to increased motor/kinetic activity, including increased head movements, potentially explaining increased shifts in the FoV. Postural instability, more prevalent in ADHD, may also drive increased head movements and shifts in FoV. However, as is proposed above for autistic individuals, shifting FoV from a foveal to peripheral perspective may also be an adaptive mechanism for stimulating peripheral vision.

A retinal/cortical hyperexcitability framework reflecting an excitatory/inhibitory (E/I) neurotransmitter imbalance may, at least partially, underpin the three factors identified by the ViPro-SR: hypersensitivity to contrast; detail focus; and peripheral vision activation (please see for details Rattray, 2025; Takarae & Sweeney, 2017). In line with this framework, altered signalling from inhibitory retinal interneurons would lead to differences in tonic and phasic signalling from retinal ganglion cells to the visual areas of the brain. Reduced tonic inhibition may cause increased response gain, increased neural noise and an amplified neural response in conditions of high visual contrast, potentially accounting for hypersensitivity to contrasting colours and patterns. Neural habituation to repeated, irrelevant stimuli may also be reduced, possibly leading to prolonged or sticky attention to objects and the detail-focused perceptual style widely reported in autism. Altered retinal excitability could further affect phasic neural activity required for coordinating the timing of interactions between neurons and for processing the temporal aspects of stimuli. As previously noted, lateral glancing/side viewing may thus be an adaptive strategy to facilitate processing of high temporal frequencies. Alternately, the three ViPro-SR factors could be integrated under a predictive coding framework (see e.g. Karvelis et al., 2018; Pellicano & Burr, 2012). If more weight is given to low-level sensory information than to prior knowledge about the world (prior distributions), this could lead to enhanced local feature detection and reduced generalisation and habituation, leading to feelings of sensory overwhelm and avoidance if persistent error signals are generated. There may be overweighting of local motion signals compared to global motion priors, leading to stimulation of peripheral vision to increase consistent motion signals and override noise. However empirical work is needed to test the utility of these frameworks for explaining the three domains captured by ViPro-SR.

Evidence towards convergent validity was present through moderate to strong correlations with other sensory measures, which persisted after correcting for several possible confounds. Stronger correlations for all groups between ViPro-SR and the SPQ total than between the ViPro-SR and AASP (visual component) may be due to the SPQ primarily measuring hypersensitivity to stimuli compared to the AASP that evaluates both hypersensitivity/avoiding and hyposensitivity/seeking behaviours. The latter are not the focus of ViPro-SR.

Discriminative validity was clearly illustrated by the highly significant differences in mean total scores between autistic/ADHD and comparison groups. Findings suggest an additive effect of being autistic and ADHD on the frequency of atypical visual behaviours/experiences, as measured by the ViPro-SR. This is in line with recent studies that report cumulative challenges and increased support needs in individuals with co-occurring autism and ADHD, compared to individuals with either NDC alone (Zablotsky et al., 2020). Although marked differences between autistic adults (+/- ADHD) and adults in the comparison group were predicted on the ViPro-SR, the high mean score for the ADHD adults was somewhat surprising. These findings suggest sensory differences may also be a key feature of ADHD and perhaps should be routinely assessed. The significant difference between ADHD and Autistic + ADHD groups in mean scores on the hypersensitivity to contrast and detail focus factors, but not on the peripheral activation subdomain, suggests overlapping yet distinct visual sensory phenotypes, which potentially reflect discrete variations in underlying biological circuitry of the visual pathway. Indeed, exploratory studies find atypical signalling from retinal neurons in both autism (Constable et al., 2020) and ADHD (Werner et al., 2020) but with distinct neural signatures (I. O. Lee et al., 2022). This could indicate condition-specific imbalances between glutamate and GABA neurotransmission across visual networks (I. O. Lee et al., 2022).

## Limitations

While our study offers a novel investigation into visual differences in neurodivergent people, it is not without limitations. Content validity was partially addressed through consultation with experts by experience and health professionals during item development; however, the qualitative feedback was not analysed using a systematic qualitative methodology. Future work would benefit from more rigorous evaluation of item relevance, comprehensibility, and comprehensiveness, using a combination of quantitative methods (e.g., content validity indices) and qualitative approaches (e.g., interviews and focus groups). This would include transparent and detailed reporting of the content validity procedures, including the coding framework, processes of theme development, and the systematic integration of feedback from both professional experts and experts by experience. Such comprehensive evaluation was not feasible in the present study and therefore constitutes a limitation. In addition, future content validity work could examine items’ correspondence and distinctiveness with respect to subjective assessment by experts of different elements of the visual processing construct (e.g., perceptual sensitivity, affective reactivity and behavioural response; see for a full discussion of these constructs, He et al., 2023), thereby clarifying how these components are represented within the ViPro-SR and their relevance to the overall construct of visual processing. Such work could be complemented by administering the scale in conjunction with objective tests of these components and examining their associations with ViPro-SR. We encourage collaborative approaches to future measurement development and invite other research teams to build on this work, particularly with respect to content validity evaluations, as comprehensive measure development is resource intensive and may be challenging to accomplish within a single research team.

Recruiting participants for a study specifically about occupational burnout may have led to sampling bias, if people experiencing work stress and burnout were more motivated to participate (e.g. to share their experiences) or less likely to take part (e.g. due to overwhelm). Future validation of ViPro-SR in a more neutrally affective context is therefore recommended. Our sample was primarily white, female and highly educated, deviating from prevailing demographic characteristics of the broader autistic or ADHD populations (Chung et al., 2019; Zeidan et al., 2022), and potentially reflecting social media-based sampling bias (Rødgaard et al., 2022). Further research is needed to determine if the measure assesses the same construct across varied cultural and educational backgrounds as well as across gender identities that are inclusive of those beyond gender binary. If the ViPro-SR is to be used outside of the UK context, then it would require adaptation to ensure its appropriateness for specific languages and/or cultural contexts and further psychometric testing in the applicable population.

Although our inclusion criteria encompassed neurodivergence in general, we primarily recruited autistic adults and adults with ADHD, and the sample sizes for adults endorsing other NDCs (such as dyslexia) were too small to be included in our analysis of diagnostic group differences. We were therefore unable to investigate discriminant validity with respect to other NDCs; standardisation in samples with a range of NDCs is warranted to determine if the measure specifically captures visual processing differences in autism and ADHD or is applicable across NDCs. In addition, the dimensionality, validity and reliability of ViPro-SR should be tested in separate groups of people with distinct traits, for example autism only or ADHD only traits, to establish whether its measurement properties differ across populations. Given mixed findings around whether phenotypic differences exist between clinically diagnosed and self-identified autism/ADHD (e.g. Banker et al., 2025; English et al., 2025), ascertaining whether the ViPro-SR measures visual processing equivalently across autism status and diagnostic groups through measurement invariance testing will be an important future step to ensure valid conclusions with regards to comparisons between these groups. Future studies should also collect current medication use; we did not gather information on pharmacological treatment which may impact visual processing. Furthermore, test-retest reliability of ViPro-SR should be assessed to establish its temporal stability, which was not feasible in the present study due to logistical constraints. Future research should address this limitation and carefully consider the choice of retest interval, which should be guided by the expected stability of the underlying construct, such that it is sufficiently short to minimise true change in visual processing while being long enough to reduce recall effects. Although visual processing may change over longer periods in autism and ADHD populations (e.g. Chen et al., 2022), it is unlikely to vary meaningfully over several weeks. Accordingly, we recommend a test–retest interval of approximately two weeks (Watson, 2004) to two months (Cattell, 1970), ideally evaluated across multiple intervals (Watson, 2004). Future research should also consider that the optimal retest interval may vary by sample characteristics, such as age; for example, older samples may require shorter retest intervals than younger ones due to changes in vision.

The 10-item visual component from the AASP, along with the subset of four items related to low registration and sensory seeking, had low internal consistency within our sample, which may be attributable to its original design — not as a standalone scale, but as part of a broader quadrant-based framework. Nonetheless, the AASP offered preliminary support for the convergent validity of ViPro-SR; the 6 items related to visual hypersensitivity demonstrated satisfactory internal consistency and showed strong correlations with our new measure.

The level of language required to comprehend the ViPro-SR questions renders it inaccessible to individuals with a severe learning disability. However, a parent/carer version of the ViPro is currently under development and may have utility for this underserved group.

Differences in visual acuity, visual field and binocular vision were not assessed in the present study and thus we were unable to determine whether these moderated the association between autism/ADHD and atypical visual behaviours. Given evidence suggests visual processing is altered across all levels of the visual pathway in both NDCs, and eyesight problems are more prevalent, future studies should explore possible associations between eyesight and subjective visual experiences. However, failure of a recent study to find a relationship between subjective and objective measures of visual sensitivity (Schulz & Stevenson, 2022) cautions against an assumption that scores on self-report sensory questionnaires closely reflect the neurobiological/psychophysical functioning of the sensory system. Accordingly, our novel ViPro-SR should, at present, be regarded as an indicator of subjective visual experience (pending any future findings of a robust association between first-hand experience and objective measures).

## Conclusions and implications

To the best of our knowledge, ViPro-SR is the first self-report questionnaire for adults to specifically measure the range of visual experiences and behaviours commonly reported in autism and ADHD. Preliminary analysis indicates ViPro-SR has robust psychometric properties. Given the brevity of the measure and its classification properties, it could have clinical utility as an adjunct to a diagnostic tool kit and help delineate specific areas of need, guiding adaptations/supports outlined in individualised education plans or workplace. For instance, tinted lenses or adjusting the contrast/brightness on computer screens might be considered for an individual with high scores on the hypersensitivity to contrast items. An individual who endorses the detail focus items may particularly benefit from a calm, uncluttered workspace that is shielded from surrounding distraction and movement. A high score on the stimulation of peripheral vision items could be viewed as a regulatory mechanism for optimising visual perception and either embraced as such or addressed with a skilled optometrist if problematic. Furthermore, ViPro-SR could be used to evaluate the effect of targeted interventions designed to support aspects of visual processing highlighted by the measure as challenging for the individual, for example to assess the impact of precision tinted lenses on sensitivity to high contrast. Supplementary Table 3 shows the final ViPro-SR formatted for presentation to participants and can be freely used.

Given the psychometric support for the multidimensionality of the ViPro-SR, which reflects three related but distinct latent constructs, the most appropriate approach is to compute subscale scores by summing the items that load on each factor. An overall 11-item total score can also be calculated by summing all items, particularly given the satisfactory internal consistency. However, this should be treated only as a composite index, that is, a pragmatic summary score, rather than as a reflection of all items measuring a single underlying construct.

As a research tool, the ViPro-SR may improve our understanding of visual sensory differences in autism, ADHD and potentially other NDCs by characterising and measuring visual sensory experiences and behaviours. Administering ViPro-SR alongside cognitive and neurobiological tests of visual processing could offer mechanistic insight into sensitivities within a sensory domain that profoundly affect quality of life.

## Supplementary Information

Below is the link to the electronic supplementary material.

Supplementary Material 1 (DOCX 1.70 MB)

## Data Availability

Due to the sensitive nature of the data that support the findings of this study, data are not publicly available.
