# Supplementary material for: Development and psychometric validation of a novel, self-report visual processing questionnaire (ViPro-SR) for neurodivergent adults
Source: Curr Psychol. 2026 Apr 28;45(9):871. doi: 10.1007/s12144-026-09409-7 (PMC13124804; doi:10.1007/s12144-026-09409-7)
Supplement: Supplementary file 1 — Supplementary Material 1 (DOCX 1.70 MB) [file 12144_2026_9409_MOESM1_ESM.docx]

Supplementary Materials

Title: Development and psychometric validation of a novel, self-report visual processing questionnaire (ViPro-SR) for neurodivergent adults

Journal name: Current Psychology

**Supplementary Table 1. Initial 55 items for the parent/carer-report visual processing questionnaire (ViPro) developed from clinical, academic and expert by experience sources** (not including responses provided on X of visual experiences that might be special to autism). Corresponding final ViPro-SR items (adapted for self-report) are included in the right-hand column of the table and italicised.

|  | **Initial 55 ViPro items** | ***Corresponding Final ViPro-SR items*** |
| --- | --- | --- |
| Item | **Theme: Hypersensitivity** | ***Final ViPro-SR Theme: Hypersensitivity to Contrast*** |
| 1. | Shows light sensitivity, e.g. covers eyes when bright lights are turned on | |
| 2. | Finds fluorescent or harsh lighting distracting or upsetting (e.g. becomes overactive or struggles to focus) | |
| 3. | Prefers dim lighting/being in the shade to bright lights/being in the sunshine | |
| 4. | Changes the brightness control on the TV to make the screen dimmer | |
| 5. | Acts calmer in dim lights | |
| 6. | Squints, narrow’s eyes or closes one eye, possibly to blur stimuli | |
| 7. | Dislikes visually busy places such as playgrounds and shopping centres | |
| 9. | Avoids eye contact | |
| 10. | Struggles to concentrate in a visually cluttered space | |
| 11. | Turns or looks away from a large amount of visual information, such as a busy picture | |
| 12. | Looks at objects with quick glances | |
| 13. | Avoids looking at stripes or busy patterns – seems to find them overwhelming | *4. Patterns or stripes look as if they are moving or flickering*  *8. Looking at stripes or busy patterns is uncomfortable for me* |
| 14. | Complains that it hurts to look at a white page or that it looks too ‘white’, shiny or bright | *6. I struggle to look at high contrast images such as black letters on a bright screen or on bright white paper* |
|  | **Theme: Hyposensitivity** | **Final ViPro-SR Theme: Peripheral vision activation** |
| 15. | Enjoys and creates flicker, for example by repetitive blinking or moving fingers or objects in front of eyes | |
| 16. | Looks for longer than is usual at light patterns, created for example by slatted blinds | |
| 17. | Can become fixated by busy patterns, e.g. on carpets | |
| 18. | Attracted to shiny surfaces or mirrors | |
| 19. | Changes the brightness control on the TV to make the screen brighter | |
| 20. | Appears to sometimes see halos, starbursts or colours around lights | |
| 21. | Stares at or through objects or people or stares into space | |
| 22. | Pushes or rubs eyes | |
| 23. | Spins objects close to face | |
| 24. | Preoccupation with spinning, rotating, flipping or twirling objects, e.g. whirling fan blades | *2. I like looking at things that spin such as a washing machine, ceiling fan, or spinning coin* |
|  | **Theme: Colour** | |
| 25. | Clearly prefers particular colours, e.g. chooses food, toys or clothes of a certain colour | |
| 26. | Avoids or shows dislike of particular colours | |
| 27. | Plays with the colour setting control on the TV | |
|  | **Theme: Depth perception and stereopsis** | |
| 28. | Struggles to judge distances so bumps into furniture | |
| 29. | Has difficulty catching balls | |
| 30. | Covers/closes an eye in order to only use one eye (issues with binocular rivalry) | |
| 31. | Tilts or turns head to focus vision | *5. I tilt or turn my head sideways to look at things* |
| 32. | Has difficulty picking things up or putting them down (e.g. may misjudge distance from own hand to table, so bump cup back down on table) | |
| 33. | Is hesitant or fearful of going up or down stairs – may need prompts | |
| 34. | Gets stuck on thresholds, for example going through doorways | |
| 35. | Demonstrates nystagmus, especially if tired of after watching a spinning object | |
|  | **Theme: Motion processing** | |
| 36. | Tries to avoid or becomes anxious around fast-moving objects | |
| 37. | Struggles to judge the speed of objects | |
| 38. | Struggles to track and follow moving objects | |
| 39. | Avoids or has trouble using revolving doors | |
| 40. | Hesitates when getting on or off moving things like an escalator | |
| 41. | Likes to watch doors open and close | |
| 42. | Finds travelling in a moving vehicle worrying or upsetting | |
| 43. | Complains or worries that things seem to be coming at them | |
| 44. | Avoids automatic doors | |
|  | **Theme: Foveal and peripheral vision (integrating visual information)** | |
| 45. | Looks out of the corner or side of their eye (shows lateral glancing) | *10. When looking at an object carefully, I hold it to the side of my face (i.e., looking at it with my peripheral vision)* |
| 46. | Plays or reaches for an object without looking at it | |
| 47. | Finds that things seem to fly apart, or images may fragment | |
|  | **Theme: Detail-focused processing style** | **Final ViPro-SR Theme: Detail focus** |
| 48. | Quick to find objects amongst a selection, such as a particular book on a bookshelf or toy in a toy box | *11. I am quick to spot things, such as a particular book on a bookshelf or an object in a drawer* |
| 49. | Shows a preference for focusing on a detail rather than the whole object | *9. I tend to focus on details or parts of objects/scenes rather than the whole thing* |
| 50. | Fixates on one object while ignoring others in the room | |
| 51. | Seems to notice tiny visual details, such as a scratch on a table | *3. I notice tiny details that other people miss, such as a thread on a patterned carpet, a tiny scratch on a table, or a slight change in font size* |
| 52. | Skilled at matching objects according to visual features such as size | |
| 53. | Quick to notice small changes in a familiar environment | *1. I am quick to notice movement ‘out of the corner of my eye,’ such as a fly in the room* |
| 54. | Excellent visual acuity – can see small details at a distance | *7. I am more likely than other people to notice things in the far distance* |
| 55. | Enjoys puzzles and completes quickly for age | |

**Supplementary Table 2. Initial 18-item ViPro-SR**

|  |  | **Never** | **Very rarely** | **Some-times** | **Often** | **Very often** | **Always** |
| --- | --- | --- | --- | --- | --- | --- | --- |
| 1. | I am sensitive to bright light e.g. I often wear sunglasses or dim indoor lights or the screen brightness on my phone, computer, or TV* |  |  |  |  |  |  |
| 2. | Bright or flickering lights make me feel anxious, exhausted, or unwell e.g., fluorescent lights or festive lights that flash on and off* |  |  |  |  |  |  |
| 3. | I don’t like some colour(s) and try to avoid them* |  |  |  |  |  |  |
| 4. | Looking at stripes or busy patterns is uncomfortable for me |  |  |  |  |  |  |
| 5. | I struggle to look at high contrast images such as black letters on a bright screen or on bright white paper |  |  |  |  |  |  |
| 6. | Patterns or stripes look as if they are moving or flickering |  |  |  |  |  |  |
| 7. | I notice tiny details that other people miss, such as a thread on a patterned carpet, a tiny scratch on a table, or a slight change in font size |  |  |  |  |  |  |
| 8. | I am quick to notice movement ‘out of the corner of my eye,’ such as a fly in the room |  |  |  |  |  |  |
| 9. | I tend to focus on details or parts of objects/scenes rather than the whole thing |  |  |  |  |  |  |
| 10. | I am more likely than other people to notice things in the far distance |  |  |  |  |  |  |
| 11. | I am quick to spot things, such as a particular book on a bookshelf or an object in a drawer |  |  |  |  |  |  |
| 12. | I like looking at patterns, such as tiles on a wall, lines on a leaf, or ripples on a pond* |  |  |  |  |  |  |
| 13. | I like to look at or to make flickering light e.g. by blinking a lot, by moving my fingers quickly in front of my eyes, or by looking at sunlight flickering through the trees* |  |  |  |  |  |  |
| 14. | When looking at an object carefully, I hold it to the side of my face (i.e., looking at it with my peripheral vision) |  |  |  |  |  |  |
| 15. | I find making eye contact uncomfortable or stressful and try to avoid this* |  |  |  |  |  |  |
| 16. | I often bump into things, knock things over or stumble on stairs* |  |  |  |  |  |  |
| 17. | I like looking at things that spin such as a washing machine, ceiling fan, or spinning coin |  |  |  |  |  |  |
| 18. | I tilt or turn my head sideways to look at things |  |  |  |  |  |  |

Note: *Items excluded from the final 11-item version

**Supplementary Table 3.** **Visual Processing Questionnaire – Self-Report Version (ViPro-SR)**

Some people struggle to process everyday sensory information – what they see, hear, smell, feel or taste. Their senses may be over- or under-sensitive, or switch between these states at different times.

The questions below ask about your processing of visual information. For each item listed below, please select the answer that best describes how often each happens for you.

|  | **Never** | **Very rarely** | **Some-times** | **Often** | **Very often** | **Always** |
| --- | --- | --- | --- | --- | --- | --- |
| 1. I am quick to notice movement ‘out of the corner of my eye,’ such as a fly in the room |  |  |  |  |  |  |
| 1. I like looking at things that spin such as a washing machine, ceiling fan, or spinning coin |  |  |  |  |  |  |
| 1. I notice tiny details that other people miss, such as a thread on a patterned carpet, a tiny scratch on a table, or a slight change in font size |  |  |  |  |  |  |
| 1. Patterns or stripes look as if they are moving or flickering |  |  |  |  |  |  |
| 1. I tilt or turn my head sideways to look at things |  |  |  |  |  |  |
| 1. I struggle to look at high contrast images such as black letters on a bright screen or on bright white paper |  |  |  |  |  |  |
| 1. I am more likely than other people to notice things in the far distance |  |  |  |  |  |  |
| 1. Looking at stripes or busy patterns is uncomfortable for me |  |  |  |  |  |  |
| 1. I tend to focus on details or parts of objects/scenes rather than the whole thing |  |  |  |  |  |  |
| 1. When looking at an object carefully, I hold it to the side of my face (i.e., looking at it with my peripheral vision) |  |  |  |  |  |  |
| 1. I am quick to spot things, such as a particular book on a bookshelf or an object in a drawer |  |  |  |  |  |  |

Scoring: Never=0; Very rarely=1; Sometimes=2; Often=3; Very often=4; Always=5. To obtain a total score, sum responses for all items. Higher scores reflect greater differences in subjective visual experiences, with a maximum score of 55. There are no reverse scored items. Psychometric findings support the use of subscale scores rather than a general total score. Where a total score is nevertheless used, it should be treated as a pragmatic composite summary rather than a representation of a single underlying construct. Subscales are comprised of the following items: Hypersensitivity to contrast (items 4, 6 & 8); Detail focus (items 1, 3, 7, 9 & 11); Peripheral vision activation (items 2, 5 & 10).

If used in paper format, please consider printing on lightly coloured paper (e.g. pale green) to reduce contrast and increase accessibility.

**Supplementary Table 4.** Sample demographic information stratified by diagnostic status

|  | | N (%) by diagnostic group | | | |
| --- | --- | --- | --- | --- | --- |
|  | | Autistic | ADHD | Autistic + ADHD | Comparison |
| Total sample n (%) | | 214 | 45 | 113 | 70 |
|  | Cis female | 137_a_ (64.02) | 36_b_ (80.00) | 71_a_ (62.83) | 54_b_ (77.14) |
|  | Cis male | 57_a_ (26.64) | 7_a,b_ (15.56) | 19_b_ (16.81) | 13_a,b_ (18.57) |
|  | ^1^ Gender diverse | 20_a_ (9.35) | 2_a_ (4.44) | 23_b_ (20.35) | 3_a_ (4.29) |
|  | Age band 18-34 years | 48_a_ (22.43) | 19_b_ (42.22) | 50_b_ (44.25) | 27_b_ (38.57) |
|  | Age band 35-54 years | 108_a_ (50.47) | 21_a_ (46.67) | 50_a_ (44.25) | 33_a_ (47.14) |
|  | Age band 55-75 years | 58_a_ (27.10) | 5_b_ (11.11) | 13_b_ (11.50) | 10_b_ (14.29) |
|  | ^2^Ethnic group - White | 195_a_ (91.98) | 31_b_ (70.45) | 101_a_ (90.18) | 60_a_ (86.96) |
|  | ^3^Education - Undergraduate degree or higher | 161_a_ (75.23) | 38_a,b_ (84.44) | 91_a,b_ (81.25) | 63_b_ (90.00) |
| RAADS-14 autistic traits, M (SD) | | 32.23_a_ (8.01) | 21.36_b_ (10.78) | 34.30_c_ (5.99) | 10.76_d_ (9.39) |
| ASRS-5 ADHD traits, M (SD) | | 11.22_a_ (3.62) | 16.07_b_ (2.73) | 15.65_b_ (3.32) | 8.23_c_ (3.69) |

Note: Values in the same row not sharing the same subscript are significantly different at *p* < .05 based on Chi-Square and Games-Howell post-hoc comparisons.

*^1^Non-binary and other self-describing gender identities (transgender woman, transgender man, genderfluid, other, prefer not say)*

*^2^English, Welsh, Scottish, Northern Irish, British, Irish, Gypsy or Irish Traveller, any other white background*

*^3^Autistic+ADHD group (n=112)*

**Supplementary Table 5.** Reported neurodivergent and mental health conditions within each group

|  | Neurodivergent groups_4 | | | |
| --- | --- | --- | --- | --- |
|  | Autistic N=214 | ADHD  N=45 | Autistic and ADHD  N=113 | ComparisonN=70 |
|  | N (%) | N (%) | N (%) | N (%) |
| Reported other neurodivergent conditions (clinically diagnosed or self-identified) | | | | |
| Dyslexia | 14 (6.5) | 9 (20.0) | 19 (16.8) | 3 (4.3) |
| Dyspraxia | 21 (9.8) | 5 (11.1) | 25 (22.1) | 1 (1.4) |
| Dyscalculia | 13 (6.1) | 2 (4.4) | 4 (3.5) | 1 (1.4) |
| Intellectual disability | 1 (0.5) | 0 (0.0) | 0 (0.0) | 0 (0.0) |
| Other^1^ | 14 (6.5) | 2 (4.4) | 13 (11.5) | 6 (8.6) |
| Reported mental health conditions | | | | |
| Major depressive disorder | 40 (18.7) | 8 (17.8) | 31 (27.4) | 10 (14.3) |
| Generalised anxiety disorder | 84 (39.3) | 16 (35.6) | 63 (55.8) | 10 (14.3) |
| Obsessive compulsive disorder | 17 (7.9) | 3 (6.7) | 11 (9.7) | 2 (2.9) |
| Post-traumatic stress disorder | 19 (8.9) | 5 (11.1) | 20 (17.7) | 3 (4.3) |
| Anorexia nervosa | 12 (5.6) | 0 (0.0) | 3 (2.7) | 0 (0.0) |
| Bulimia nervosa | 5 (2.3) | 1 (2.2) | 3 (2.7) | 3 (4.3) |
| Avoidant restrictive food intake disorder | 5 (2.3) | 0 (0.0) | 6 (5.3) | 0 (0.0) |
| Binge eating disorder | 6 (2.8) | 0 (0.0) | 4 (3.5) | 3 (4.3) |
| Borderline personality disorder | 8 (3.7) | 3 (6.7) | 4 (3.5) | 0 (0.0) |
| Other | 28 (13.1) | 5 (11.1) | 19 (16.8) | 2 (2.9) |
| Reported not having any of the above mental health conditions | 93 (43.5) | 21 (46.7) | 26 (23.0) | 50 (71.4) |
| Prefer not to say (mental health) | 2 (0.9) | 1 (2.2) | 1 (0.9) | 1 (1.4) |

Note: ^1^Other represents where participants indicated having a neurodivergent condition that was different from those specified in the survey (i.e. autism, ADHD, dyslexia, dyscalculia and intellectual disability). Corresponding open text responses included a wide range of physical, neurodivergent and mental health conditions under the category ‘Other’.

**Supplementary Table 6**. ViPro-SR items included in the final 11-item 3-factor solution

| **Item** | **ViPro-SR statement in full** |
| --- | --- |
| *Factor 1. Hypersensitivity to contrast* | |
| I04. | Looking at stripes or busy patterns is uncomfortable for me |
| I05. | I struggle to look at high contrast images such as black letters on a bright screen or on bright white paper |
| I06. | Patterns or stripes look as if they are moving or flickering |
| *Factor 2. Detail focus* | |
| I07. | I notice tiny details that other people miss, such as a thread on a patterned carpet, a tiny scratch on a table, or a slight change in font size |
| I08. | I am quick to notice movement ‘out of the corner of my eye,’ such as a fly in the room |
| I09. | I tend to focus on details or parts of objects/scenes rather than the whole thing |
| I10. | I am more likely than other people to notice things in the far distance |
| I11. | I am quick to spot things, such as a particular book on a bookshelf or an object in a drawer |
| *Factor 3. Peripheral vision activation* | |
| I14. | When looking at an object carefully, I hold it to the side of my face (i.e., looking at it with my peripheral vision) |
| I17. | I like looking at things that spin such as a washing machine, ceiling fan, or spinning coin |
| I18. | I tilt or turn my head sideways to look at things |

| **Supplementary Table 7.** Item stability of ViPro-SR items across 1,000 bootstrap samples. | | | | | |
| --- | --- | --- | --- | --- | --- |
| **Item** | **Label** | **Empirical factor** | **Factor 1** | **Factor 2** | **Factor 3** |
|  |  |  | *Hypersensitivity to contrast* | *Detail focus* | *Peripheral vision activation* |
| I04 | Stripes or busy patterns uncomfortable | 1 | 1 | 0 | 0 |
| I05 | Struggle with high contrast images | 1 | 1 | 0 | 0 |
| I06 | Patterns moving or flickering | 1 | 1 | 0 | 0 |
| I07 | Notice tiny details | 2 | 0 | 1 | 0 |
| I08 | Notice movement | 2 | 0 | 1 | 0 |
| I09 | Focus on details | 2 | 0 | 1 | 0 |
| I10 | Notice far distance | 2 | 0 | 1 | 0 |
| I11 | Quick to spot things | 2 | 0 | 1 | 0 |
| I14 | Looking with peripheral vision | 3 | 0.001 | 0 | 0.999 |
| I17 | Looking at things that spin | 3 | 0.001 | 0 | 0.999 |
| I18 | Tilt or turn head | 3 | 0.001 | 0 | 0.999 |

**Supplementary Table 8.** ViPro-SR (11-item) total score descriptive statistics and ViPro-SR total score correlations with SPQ35 total score, AASP visual component total score, AASP visual hypersensitivity items (sensory sensitivity and avoidance) and AASP visual hyposensitivity items (low registration and sensory seeking) within diagnostic groups

|  | |  | Correlations | | | |
| --- | --- | --- | --- | --- | --- | --- |
|  | |  | ViPro-SR total correlation (by group) with | | | |
| Neurotype groups (clinical diagnosis and self-identified) | | ViPro-SR total score  Mean(SD) | SPQ 35 total | AASP visual component total | AASP visual hypersensitivity items | AASP visual hyposensitivity items |
|  | Autistic (n=214) | 26.73 (8.89) | -0.64*** | 0.55*** (0.82) | 0.67*** | -0.12 (-0.31) |
|  | ADHD (n=45) | 23.40 (8.90) | -0.78*** | 0.37* (0.59) | 0.56*** | -0.46** (≈-1) |
|  | Autistic and ADHD (n=113) | 28.87 (7.61) | -0.64*** | 0.50***^a^ (0.76) | 0.61*** | 0.09 (0.18) |
|  | Comparison (n=70) | 14.97 (6.87) | -0.52*** | 0.42*** (0.59) | 0.50*** | 0.01 (0.02) |

Note: **p* < .05. ***p* < .01. ****p* < .001. ^a^*n* = 111 due to one missing response. Disattenuated correlations (α) in parentheses.

**Supplementary Table 9.** Correlations between ViPro-SR factor scores and SPQ-35 and AASP total scores with 95% CI

|  |  | 1 | 2 | 3 | 4 |
| --- | --- | --- | --- | --- | --- |
|  | ViPro-SR Factor 1 (Hypersensitivity to contrast) | 1 |  |  |  |
|  | ViPro-SR Factor 2  (Detail focus) | .43^***^  [0.35, 0.51] | 1 |  |  |
|  | ViPro-SR Factor 3  (Peripheral vision activation) | .40^***^  [0.32, 0.47] | .374^***^  [0.29, 0.45] | 1 |  |
|  | SPQ-35 total score | -.52^***^  [-0.58, -0.44] | -.66^***^  [-0.71, -0.61] | -.40^***^  [-0.48, -0.32] | 1 |
|  | AASP total score | .50^***^ (.70)  [0.43, 0.57] | .45^***^ (.63)  [0.37, 0.52] | .40^***^ (.64)  [0.32, 0.48] | -.45^***^  [-0.52, -0.37] |

Note: ***. Correlation is significant at the 0.001 level (2-tailed). 95% CI in squared brackets. Disattenuated correlations (α) in parentheses.

**Supplementary Figure 1**


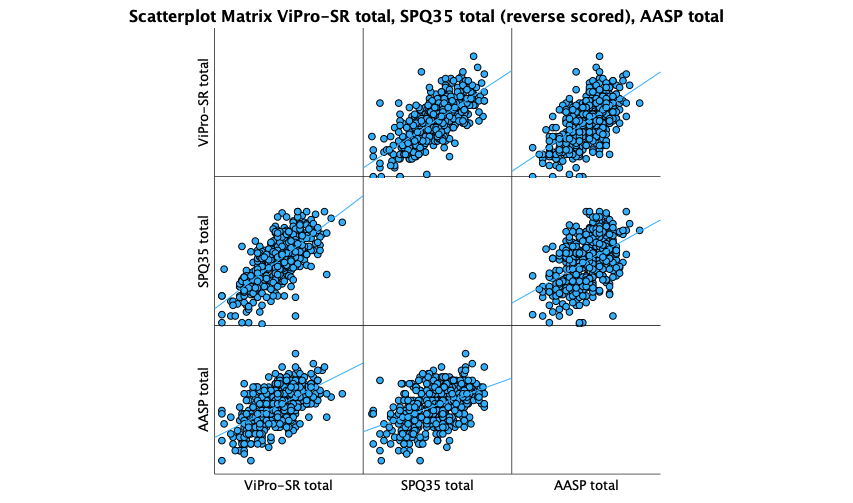


**Supplementary References**

Jöreskog, K. G. (1971). Simultaneous factor analysis in several populations. *Psychometrika*, *36*(4), 409-426. <https://doi.org/10.1007/BF02291366>

Josyfon, E., Spain, D., Blackmore, C., Murphy, D., & Oakley, B. (2023). Alexithymia in Adult Autism Clinic Service-Users: Relationships with Sensory Processing Differences and Mental Health. *Healthcare (Basel)*, *11*(24). <https://doi.org/10.3390/healthcare11243114>

Jurek, L., Duchier, A., Gauld, C., Hénault, L., Giroudon, C., Fourneret, P., Cortese, S., & Nourredine, M. (2025). Sensory Processing in Individuals With Attention-Deficit/Hyperactivity Disorder Compared With Control Populations: A Systematic Review and Meta-Analysis. *Journal of the American Academy of Child & Adolescent Psychiatry*, *64*(10), 1132-1147. <https://doi.org/10.1016/j.jaac.2025.02.019>

Kaiser, H. F. (1960). The application of electronic computers to factor analysis. *Educational and psychological measurement*, *20*(1), 141-151.

Kaiser, H. J. (1970). A second generation little jiffy. *Psychometrika 35*(4), 401-415.

Kaiser, H. J. a. R., J. . (1974). Little jiffy, mark iv. *Educational and psychological measurement*, *34*(1), 111-117.

Karvelis, P., Seitz, A. R., Lawrie, S. M., & Seriès, P. (2018). Autistic traits, but not schizotypy, predict increased weighting of sensory information in Bayesian visual integration. *Elife*, *7*. <https://doi.org/10.7554/eLife.34115>

Khanahmadi, S., Sourtiji, H., Khanahmadi, Z., & Sheikhtaheri, A. (2023). Effect of a sensory diet smartphone application on the symptoms of children with attention deficit hyperactivity disorder (ADHD): A feasibility study. *Heliyon*, *9*(8), e19086. <https://doi.org/10.1016/j.heliyon.2023.e19086>

Kline, R. B. (2016). *Principles and practice of structural equation modelling* (4th ed.). Guilford Press.

Kooij, J. J., & Bijlenga, D. (2014). High prevalence of self-reported photophobia in adult ADHD. *Front Neurol*, *5*, 256. <https://doi.org/10.3389/fneur.2014.00256>

Koopmans, E., & Schiller, D. C. (2022). Understanding Causation in Healthcare: An Introduction to Critical Realism. *Qualitative Health Research*, *32*(8-9), 1207-1214. <https://doi.org/10.1177/10497323221105737>

Kröger, A., Hof, K., Krick, C., Siniatchkin, M., Jarczok, T., Freitag, C. M., & Bender, S. (2014). Visual processing of biological motion in children and adolescents with attention-deficit/hyperactivity disorder: an event related potential-study. *PLoS One*, *9*(2), e88585. <https://doi.org/10.1371/journal.pone.0088585>

Kyriazos, T. (2018). Applied Psychometrics: Sample Size and Sample Power Considerations in Factor Analysis (EFA, CFA) and SEM in General. *Psychology*, *09*, 2207-2230. <https://doi.org/10.4236/psych.2018.98126>

Lane, S. J., Leão, M. A., & Spielmann, V. (2022). Sleep, Sensory Integration/Processing, and Autism: A Scoping Review. *Front Psychol*, *13*, 877527. <https://doi.org/10.3389/fpsyg.2022.877527>

Larson, A. M., & Loschky, L. C. (2009). The contributions of central versus peripheral vision to scene gist recognition. *J Vis*, *9*(10), 6 1-16. <https://doi.org/10.1167/9.10.6>

Lauritzen, S. L. (1996). *Graphical Models*. Clarendon Press.

Lee, E.-H., Lee, Y. W., Lee, K.-W., Kim, H. J., Hong, S., Kim, S. H., & Kang, E. H. (2022). Development and psychometric evaluation of a new brief scale to measure eHealth literacy in people with type 2 diabetes. *BMC Nursing*, *21*(1), 297. <https://doi.org/10.1186/s12912-022-01062-2>

Lee, I. O., Skuse, D. H., Constable, P. A., Marmolejo-Ramos, F., Olsen, L. R., & Thompson, D. A. (2022). The electroretinogram b-wave amplitude: a differential physiological measure for Attention Deficit Hyperactivity Disorder and Autism Spectrum Disorder. *J Neurodev Disord*, *14*(1), 30. <https://doi.org/10.1186/s11689-022-09440-2>

Lee, T.-Y., Tsai, S.-J., Chen, T.-J., & Chen, M.-H. (2021). Risk of migraine development among children and adolescents with autism spectrum disorder: A nationwide longitudinal study. *Research in Autism Spectrum Disorders*, *89*, 101880. <https://doi.org/https://doi.org/10.1016/j.rasd.2021.101880>

Leekam, S. R., Nieto, C., Libby, S. J., Wing, L., & Gould, J. (2007). Describing the sensory abnormalities of children and adults with autism. *J Autism Dev Disord*, *37*(5), 894-910. <https://doi.org/10.1007/s10803-006-0218-7>

Lim, S., & Jahng, S. (2019). Determining the number of factors using parallel analysis and its recent variants. *Psychol Methods*, *24*(4), 452-467. <https://doi.org/10.1037/met0000230>

Little, J. A. (2018). Vision in children with autism spectrum disorder: a critical review. *Clin Exp Optom*. <https://doi.org/10.1111/cxo.12651>

Little, L. M., Dean, E., Tomchek, S., & Dunn, W. (2018). Sensory Processing Patterns in Autism, Attention Deficit Hyperactivity Disorder, and Typical Development. *Phys Occup Ther Pediatr*, *38*(3), 243-254. <https://doi.org/10.1080/01942638.2017.1390809>

Ludlow, A. K., & Wilkins, A. J. (2009). Case report: color as a therapeutic intervention. *J Autism Dev Disord*, *39*(5), 815-818. <https://doi.org/10.1007/s10803-008-0672-5>

Lukmanji, S., Manji, S. A., Kadhim, S., Sauro, K. M., Wirrell, E. C., Kwon, C.-S., & Jetté, N. (2019). The co-occurrence of epilepsy and autism: A systematic review. *Epilepsy & Behavior*, *98*, 238-248. <https://doi.org/https://doi.org/10.1016/j.yebeh.2019.07.037>

Lynn, A. C., Padmanabhan, A., Simmonds, D., Foran, W., Hallquist, M. N., Luna, B., & O'Hearn, K. (2018). Functional connectivity differences in autism during face and car recognition: underconnectivity and atypical age-related changes. *Dev Sci*, *21*(1). <https://doi.org/10.1111/desc.12508>

MacLennan, K., O’Brien, S., & Tavassoli, T. (2022). In Our Own Words: The Complex Sensory Experiences of Autistic Adults. *Journal of Autism and Developmental Disorders*, *52*(7), 3061-3075. <https://doi.org/10.1007/s10803-021-05186-3>

MacLennan, K., Roach, L., & Tavassoli, T. (2020). The Relationship Between Sensory Reactivity Differences and Anxiety Subtypes in Autistic Children. *Autism Res*, *13*(5), 785-795. <https://doi.org/10.1002/aur.2259>

Mangalmurti, A., Kistler, W. D., Quarrie, B., Sharp, W., Persky, S., & Shaw, P. (2020). Using virtual reality to define the mechanisms linking symptoms with cognitive deficits in attention deficit hyperactivity disorder. *Scientific Reports*, *10*(1), 529. <https://doi.org/10.1038/s41598-019-56936-4>

Mardia, K. V. (1970). Measures of multivariate skewness and kurtosis with applications. *Biometrika*, *57*(3), 519-530. <https://doi.org/10.1093/biomet/57.3.519>

McDonald, R. P. (1999). *Test Theory: A Unified Treatment* (1st ed.). Psychology Press. <https://doi.org/https://doi.org/10.4324/9781410601087>

Miller, M., Sun, S., Iosif, A. M., Young, G. S., Belding, A., Tubbs, A., & Ozonoff, S. (2021). Repetitive behavior with objects in infants developing autism predicts diagnosis and later social behavior as early as 9 months. *J Abnorm Psychol*, *130*(6), 665-675. <https://doi.org/10.1037/abn0000692>

Mottron, L., Dawson, M., Soulieres, I., Hubert, B., & Burack, J. (2006). Enhanced perceptual functioning in autism: an update, and eight principles of autistic perception. *J Autism Dev Disord*, *36*(1), 27-43. <https://doi.org/10.1007/s10803-005-0040-7>

Muthén, L. K., & Muthén, B. O. (1998-2017). *Mplus User’s Guide* (Eighth ed.). Muthén & Muthén

Nahm, F. S. (2022). Receiver operating characteristic curve: overview and practical use for clinicians. *Korean J Anesthesiol*, *75*(1), 25-36. <https://doi.org/10.4097/kja.21209>

Neufeld, J., Hagström, A., Van't Westeinde, A., Lundin, K., Cauvet, É., Willfors, C., Isaksson, J., Lichtenstein, P., & Bölte, S. (2020). Global and local visual processing in autism - a co-twin-control study. *J Child Psychol Psychiatry*, *61*(4), 470-479. <https://doi.org/10.1111/jcpp.13120>

O'Nions, E., Petersen, I., Buckman, J. E. J., Charlton, R., Cooper, C., Corbett, A., Happé, F., et al. (2023). Autism in England: assessing underdiagnosis in a population-based cohort study of prospectively collected primary care data. *Lancet Reg Health Eur*, *29*, 100626. <https://doi.org/10.1016/j.lanepe.2023.100626>

Ozonoff, S., Macari, S., Young, G. S., Goldring, S., Thompson, M., & Rogers, S. J. (2008). Atypical object exploration at 12 months of age is associated with autism in a prospective sample. *Autism*, *12*(5), 457-472. <https://doi.org/10.1177/1362361308096402>

Panagiotidi, M., Overton, P. G., & Stafford, T. (2018). The relationship between ADHD traits and sensory sensitivity in the general population. *Comprehensive Psychiatry*, *80*, 179-185. <https://doi.org/https://doi.org/10.1016/j.comppsych.2017.10.008>

Parmar, K. R., Porter, C. S., Dickinson, C. M., Pelham, J., Baimbridge, P., & Gowen, E. (2021). Visual Sensory Experiences From the Viewpoint of Autistic Adults. *Front Psychol*, *12*, 633037. <https://doi.org/10.3389/fpsyg.2021.633037>

Passarello, N., Tarantino, V., Chirico, A., Menghini, D., Costanzo, F., Sorrentino, P., Fucà, E., et al. (2022). Sensory Processing Disorders in Children and Adolescents: Taking Stock of Assessment and Novel Therapeutic Tools. *Brain Sci*, *12*(11). <https://doi.org/10.3390/brainsci12111478>

Pearson. (2019). *Adolescent/Adult Sensory Profile Technical Report*. P. Education.

Peli, E., Apfelbaum, H., Berson, E. L., & Goldstein, R. B. (2016). The risk of pedestrian collisions with peripheral visual field loss. *J Vis*, *16*(15), 5. <https://doi.org/10.1167/16.15.5>

Pellicano, E., & Burr, D. (2012). When the world becomes ‘too real’: a Bayesian explanation of autistic perception. *Trends in Cognitive Sciences*, *16*(10), 504-510. <https://doi.org/https://doi.org/10.1016/j.tics.2012.08.009>

Perna, J., Bellato, A., Ganapathy, P. S., Solmi, M., Zampieri, A., Faraone, S. V., & Cortese, S. (2023). Association between Autism Spectrum Disorder (ASD) and vision problems. A systematic review and meta-analysis. *Mol Psychiatry*. <https://doi.org/10.1038/s41380-023-02143-7>

Pons, P., & Latapy, M. (2006). Computing Communities in Large Networks Using Random Walks. *J. Graph Algorithms Appl.*, *10*, 191-218. <https://doi.org/10.7155/jgaa.00124>

Proudfoot, K. (2023). Inductive/Deductive Hybrid Thematic Analysis in Mixed Methods Research. *Journal of Mixed Methods Research*, *17*(3), 308-326. <https://doi.org/10.1177/15586898221126816>

Puts, N. A., Ryan, M., Oeltzschner, G., Horska, A., Edden, R. A., & Mahone, E. M. (2020). Reduced striatal GABA in unmedicated children with ADHD at 7T. *Psychiatry Research: Neuroimaging*, *301*, 111082.

Rattray, F. (2025). *A multimethod exploration of visual processing and visual attention in autism and ADHD* King’s College London ].

Rattray, F., Ruane, M., Saliko, N., Absoud, M., & Happé, F. (2025). Gathering autistic adults’ visual experiences to inform adaptations: a qualitative interview study. *Disability & Society*, 1-28. <https://doi.org/10.1080/09687599.2025.2498415>

Redondo, B., Molina, R., Cano-Rodríguez, A., Vera, J., García, J. A., Muñoz-Hoyos, A., & Jiménez, R. (2019). Visual Perceptual Skills in Attention-deficit/Hyperactivity Disorder Children: The Mediating Role of Comorbidities. *Optom Vis Sci*, *96*(9), 655-663. <https://doi.org/10.1097/opx.0000000000001416>

Robertson, A. E., & Simmons, D. R. (2015). The Sensory Experiences of Adults with Autism Spectrum Disorder: A Qualitative Analysis. *Perception*, *44*(5), 569-586. <https://doi.org/10.1068/p7833>

Robertson, C. E., Thomas, C., Kravitz, D. J., Wallace, G. L., Baron-Cohen, S., Martin, A., & Baker, C. I. (2014). Global motion perception deficits in autism are reflected as early as primary visual cortex. *Brain*, *137*(Pt 9), 2588-2599. <https://doi.org/10.1093/brain/awu189>

Rødgaard, E. M., Jensen, K., Miskowiak, K. W., & Mottron, L. (2022). Representativeness of autistic samples in studies recruiting through social media. *Autism Res*, *15*(8), 1447-1456. <https://doi.org/10.1002/aur.2777>

Scheerer, N. E., Pourtousi, A., Yang, C., Ding, Z., Stojanoski, B., Anagnostou, E., Nicolson, R., et al. (2024). Transdiagnostic Patterns of Sensory Processing in Autism and ADHD. *Journal of Autism and Developmental Disorders*, *54*(1), 280-292. <https://doi.org/10.1007/s10803-022-05798-3>

Schulz, S. E., & Stevenson, R. A. (2022). Convergent Validity of Behavioural and Subjective Sensitivity in Relation to Autistic Traits. *J Autism Dev Disord*, *52*(2), 758-770. <https://doi.org/10.1007/s10803-021-04974-1>

Simmons, D. R., Robertson, A. E., McKay, L. S., Toal, E., McAleer, P., & Pollick, F. E. (2009). Vision in autism spectrum disorders. *Vision Res*, *49*(22), 2705-2739. <https://doi.org/10.1016/j.visres.2009.08.005>

Sinha, R., Hoon, M., Baudin, J., Okawa, H., Wong, R. O. L., & Rieke, F. (2017). Cellular and Circuit Mechanisms Shaping the Perceptual Properties of the Primate Fovea. *Cell*, *168*(3), 413-426 e412. <https://doi.org/10.1016/j.cell.2017.01.005>

Skocic, D., Brown, T., Yu, M.-L., & Reed, K. Convergent validity of two adult self-report sensory scales: Comparing the Adolescent/Adult Sensory Profile and the Sensory Processing Measure 2–Adult Form. *Australian Occupational Therapy Journal*, *n/a*(n/a). <https://doi.org/https://doi.org/10.1111/1440-1630.12963>

Soltan, M. H., Albalawi, R. A., Alnawmasi, N. S. M., Alshammari, W. F. D., AlOmari, L. H., Ibrahim, M. I. F. B., Alshammari, S. H. M., et al. (2023). Association between Migraine and Attention Deficit Hyperactivity Disorder: Systematic Review. *Pharmacophore*, *14*(2), 58-64. <https://doi.org/10.51847/wSrSsrseWK>

Song, Y., Hakoda, Y., Sanefuji, W., & Cheng, C. (2015). Can They See It? The Functional Field of View Is Narrower in Individuals with Autism Spectrum Disorder. *PLoS One*, *10*(7), e0133237. <https://doi.org/10.1371/journal.pone.0133237>

Steiger, J. H. (1990). Structural Model Evaluation and Modification: An Interval Estimation Approach. *Multivariate Behavioral Research*, *25*(2), 173-180. <https://doi.org/10.1207/s15327906mbr2502_4>

Suzuki, K., Suzuki, S., Shiina, T., Okamura, M., Haruyama, Y., Tatsumoto, M., & Hirata, K. (2021). Investigating the relationships between the burden of multiple sensory hypersensitivity symptoms and headache-related disability in patents with migraine. *The Journal of Headache and Pain*, *22*(1), 77. <https://doi.org/10.1186/s10194-021-01294-8>

Syu, Y. C., & Lin, L. Y. (2018). Sensory Overresponsivity, Loneliness, and Anxiety in Taiwanese Adults with Autism Spectrum Disorder. *Occup Ther Int*, *2018*, 9165978. <https://doi.org/10.1155/2018/9165978>

Tabachnick, B. G., Fidell, L. S., & Ullman, J. B. (2013). *Using multivariate statistics* (Vol. 6). Pearson

Takarae, Y., & Sweeney, J. (2017). Neural Hyperexcitability in Autism Spectrum Disorders. *Brain Sci*, *7*(10). <https://doi.org/10.3390/brainsci7100129>

Tavassoli, T., Hoekstra, R. A., & Baron-Cohen, S. (2014). The Sensory Perception Quotient (SPQ): development and validation of a new sensory questionnaire for adults with and without autism. *Mol Autism*, *5*, 29. <https://doi.org/10.1186/2040-2392-5-29>

Taylor, E., Holt, R., Tavassoli, T., Ashwin, C., & Baron-Cohen, S. (2020). Revised scored Sensory Perception Quotient reveals sensory hypersensitivity in women with autism. *Mol Autism*, *11*(1), 18. <https://doi.org/10.1186/s13229-019-0289-x>

Treister, R., Eisenberg, E., Demeter, N., & Pud, D. (2015). Alterations in Pain Response are Partially Reversed by Methylphenidate (Ritalin) in Adults with Attention Deficit Hyperactivity Disorder (ADHD). *Pain Practice*, *15*(1), 4-11. <https://doi.org/https://doi.org/10.1111/papr.12129>

Tucker, L. R., & Lewis, C. (1973). A reliability coefficient for maximum likelihood factor analysis. *Psychometrika*, *38*(1), 1-10. <https://doi.org/10.1007/BF02291170>

Turano, K., Herdman, S. J., & Dagnelie, G. (1993). Visual stabilization of posture in retinitis pigmentosa and in artificially restricted visual fields. *Invest Ophthalmol Vis Sci*, *34*(10), 3004-3010.

Turano, K., & Wang, X. (1992). Motion thresholds in retinitis pigmentosa. *Invest Ophthalmol Vis Sci*, *33*(8), 2411-2422.

Unal, I. (2017). Defining an Optimal Cut-Point Value in ROC Analysis: An Alternative Approach. *Comput Math Methods Med*, *2017*, 3762651. <https://doi.org/10.1155/2017/3762651>

Ustun, B., Adler, L. A., Rudin, C., Faraone, S. V., Spencer, T. J., Berglund, P., Gruber, M. J., & Kessler, R. C. (2017). The World Health Organization Adult Attention-Deficit/Hyperactivity Disorder Self-Report Screening Scale for DSM-5. *JAMA Psychiatry*, *74*(5), 520-527. <https://doi.org/10.1001/jamapsychiatry.2017.0298>

Van der Hallen, R., Evers, K., Brewaeys, K., Van den Noortgate, W., & Wagemans, J. (2015). Global processing takes time: A meta-analysis on local-global visual processing in ASD. *Psychological Bulletin*, *141*(3), 549-573. <https://doi.org/10.1037/bul0000004>

Vater, C., Wolfe, B., & Rosenholtz, R. (2022). Peripheral vision in real-world tasks: A systematic review. *Psychonomic Bulletin & Review*, *29*(5), 1531-1557. <https://doi.org/10.3758/s13423-022-02117-w>

Vitoratou, S., Uglik-Marucha, E., Hayes, C., Pickles, A. . (2023). *A comprehensive guide for assessing measurement tool quality: The contemporary psychometrics (ConPsy) checklist [Withdrawn].* <https://psyarxiv.com/t2pbj/>

Wainstein, G., Rojas-Líbano, D., Crossley, N. A., Carrasco, X., Aboitiz, F., & Ossandón, T. (2017). Pupil Size Tracks Attentional Performance In Attention-Deficit/Hyperactivity Disorder. *Scientific Reports*, *7*(1), 8228. <https://doi.org/10.1038/s41598-017-08246-w>

Wang, S., Yao, B., Zhang, H., Xia, L., Yu, S., Peng, X., Xiang, D., & Liu, Z. (2023). Comorbidity of epilepsy and attention-deficit/hyperactivity disorder: a systematic review and meta-analysis. *Journal of Neurology*, *270*(9), 4201-4213. <https://doi.org/10.1007/s00415-023-11794-z>

Watson, D. (2004). Stability versus change, dependability versus error: Issues in the assessment of personality over time. *Journal of Research in Personality*, *38*(4), 319-350. <https://doi.org/https://doi.org/10.1016/j.jrp.2004.03.001>

Werner, A. L., Tebartz van Elst, L., Ebert, D., Friedel, E., Bubl, A., Clement, H. W., Lukacin, R., Bach, M., & Bubl, E. (2020). Normalization of increased retinal background noise after ADHD treatment: A neuronal correlate. *Schizophr Res*, *219*, 77-83. <https://doi.org/10.1016/j.schres.2019.04.013>

Widaman, K., & Reise, S. (1997). Exploring the measurement invariance of psychological instruments: Applications in the substance use domain. <https://doi.org/10.1037/10222-009>

Zablotsky, B., Bramlett, M. D., & Blumberg, S. J. (2020). The Co-Occurrence of Autism Spectrum Disorder in Children With ADHD. *Journal of Attention Disorders*, *24*(1), 94-103. <https://doi.org/10.1177/1087054717713638>

Zachi, E. C., Costa, T. L., Barboni, M. T. S., Costa, M. F., Bonci, D. M. O., & Ventura, D. F. (2017). Color Vision Losses in Autism Spectrum Disorders. *Front Psychol*, *8*, 1127. <https://doi.org/10.3389/fpsyg.2017.01127>

Zeidan, J., Fombonne, E., Scorah, J., Ibrahim, A., Durkin, M. S., Saxena, S., Yusuf, A., Shih, A., & Elsabbagh, M. (2022). Global prevalence of autism: A systematic review update. *Autism Research*, *15*(5), 778-790. <https://doi.org/https://doi.org/10.1002/aur.2696>
